# Spatiotemporally Explicit Epidemic Model for West Nile Virus Outbreak in Germany: An Inversely Calibrated Approach

**DOI:** 10.1007/s44197-024-00254-0

**Published:** 2024-07-04

**Authors:** Oliver Chinonso Mbaoma, Stephanie Margarete Thomas, Carl Beierkuhnlein

**Affiliations:** 1 Department of Biogeography, University of Bayreuth, Universitaetsstr. 30, 95447 Bayreuth, Germany; 2 Bayreuth Center of Ecology and Environmental Research, BayCEER, University of Bayreuth, Universitaetsstr. 30, 95447 Bayreuth, Germany; 3 Geographical Institute of the University of Bayreuth, GIB, Universitaetsstr. 30, 95447 Bayreuth, Germany; 4 Departamento de Botánico, Universidad de Granada, 18071 Granada, Spain

**Keywords:** West Nile, Mosquito-borne diseases, Inverse calibration, Epidemiological model, Population model, Mechanistic model

## Abstract

Since the first autochthonous transmission of West Nile Virus was detected in Germany (WNV) in 2018, it has become endemic in several parts of the country and is continuing to spread due to the attainment of a suitable environment for vector occurrence and pathogen transmission. Increasing temperature associated with a changing climate has been identified as a potential driver of mosquito-borne disease in temperate regions. This scenario justifies the need for the development of a spatially and temporarily explicit model that describes the dynamics of WNV transmission in Germany. In this study, we developed a process-based mechanistic epidemic model driven by environmental and epidemiological data. Functional traits of mosquitoes and birds of interest were used to parameterize our compartmental model appropriately. Air temperature, precipitation, and relative humidity were the key climatic forcings used to replicate the fundamental niche responsible for supporting mosquito population and infection transmission risks in the study area. An inverse calibration method was used to optimize our parameter selection. Our model was able to generate spatially and temporally explicit basic reproductive number (R_0_) maps showing dynamics of the WNV occurrences across Germany, which was strongly associated with the deviation from daily means of climatic forcings, signaling the impact of a changing climate in vector-borne disease dynamics. Epidemiological data for human infections sourced from Robert Koch Institute and animal cases collected from the Animal Diseases Information System (TSIS) of the Friedrich-Loeffler-Institute were used to validate model-simulated transmission rates. From our results, it was evident that West Nile Virus is likely to spread towards the western parts of Germany with the rapid attainment of environmental suitability for vector mosquitoes and amplifying host birds, especially short-distance migratory birds. Locations with high risk of WNV outbreak (Baden-Württemberg, Bavaria, Berlin, Brandenburg, Hamburg, North Rhine-Westphalia, Rhineland-Palatinate, Saarland, Saxony-Anhalt and Saxony) were shown on R_0_ maps. This study presents a path for developing an early warning system for vector-borne diseases driven by climate change.

**Supplementary Information:**

The online version contains supplementary material available at 10.1007/s44197-024-00254-0.

## Introduction

### Background

West Nile Virus (WNV) is an arbovirus transmitted by mosquitoes between humans and animals [1]. WNV fever, associated with WNV infection, is widely considered a public health emergency with seasonal occurrence [2]. Due to its novel appearance, poor-surveillance, and co-circulation with other arboviruses, missing diagnosis is likely to occur, as has been reported for South America [3]. Although originally detected in Uganda, WNV has now been found on all continents and was serologically identified in Europe as far back as 1958. The pathogen is suspected to break-out when migratory bird and mosquito populations coexist in late summer [4].

WNV epidemiology varies across geographical locations with uncertainties due to prevailing local environmental and climatic conditions that alter vector and host distributions [5, 6]. Screening of human, bird, horse, and mosquito populations shows that WNV appears seasonally in Europe [7–9]. However, sudden outbreaks of WNV and their geographical patterns are not fully understood. This would be an important step towards ensuring both improved surveillance and control of WNV through an early warning system.

In 2018, 2083 autochthonous cases of WNV were reported in Europe [10]. This represented a substantial increase compared to previous years. It is assumed that optimal weather conditions for vector occurrence, abundance, survival, and extrinsic virus replication are responsible [11]. In the same year, first evidence of autochthonous transmission was detected in Germany, with additional instances in wild and domestic birds as well as equids [11]. Transmission of the virus between infected mosquitoes and host species has remained active till date, with the first human case recorded in 2019 and the first fatal case occurring in 2020 [12].

### The Role of Climate, Mosquito Dynamics, and Bird Populations in WNV Transmission

Change and variability in climate events are regarded as a potential driver of vector-borne diseases in temperate regions. Events that increase ambient temperature have been identified as a stimulant for favorable bioclimate for infectious disease vectors in Europe. Since WNV is transmitted by ectotherms that thrive in warm environments, variability in climate conditions tends to support and stabilize their establishment. Furthermore, studies have established that transmission risks and force of infection increases with respect to suitable environmental conditions created by a changing climate.

Mosquitoes are ectotherms whose occurrence, abundance, and subsequent survival are underpinned by ambient temperature and other climatic variables, particularly relative humidity and precipitation. Divergence from the mean values of these variables has been identified as the key factor responsible for the altered range and distribution of mosquito-borne diseases in temperate regions. This can also lead to the emergence or re-emergence of mosquito-borne diseases where they were otherwise absent.

Although WNV has been found in over 60 species of mosquitoes, vector competence differs across species, biotypes, and geographical location [13]. Feeding pattern, host preference, and virus replication rate are key determinants of vector capacity across mosquito species. *Culex* mosquitoes comprising *Cx. pipiens*, *Cx. molestus*, C*x. restuans* and *Cx. torrentium* have been identified as the most important vectors [14, 15]. Although *Cx. pipiens* biotypes, which are primarily ornithophilic, are important in maintaining the natural WNV enzootic cycle, their hybrids are also important in explaining the spillover of WNV to humans due to their preference for feeding on mammals [16]. Mosquitoes become infected after biting an infected bird, which has developed viremia [17]. The infected mosquito then remains infectious and can transmit the virus to humans as well as animals, such as birds and horses. Humans and horses are dead-end hosts, unable to amplify infection or facilitate cross-infection.

Birds are considered the most important host of WNV due to the multiple roles they play in maintaining an enzootic transmission cycle of WNV in nature [18]. Wild migratory birds have been identified as biological vehicles for distribution of important microbial pathogens including flaviviruses like WNV [19]. Between 2009 and 2011, serum samples obtained in Germany from 364 migratory and residential birds, 1119 domestic poultry, and 1282 horses were analyzed. WNV antibodies were identified exclusively in the migratory birds [20].

Migratory birds may be responsible for the introduction of arboviruses to new areas along their migratory route across long distances [8]. Wild birds roam freely with a high possibility of being bitten by different mosquitoes at some point in their lifetime. Understanding this process is essential for establishing an early warning system. Molecular prevalence and bird mortality, identified as key indicators of reservoir host for WNV, have been found to be to be high in birds from the Passeriformes, Charadriiformes, Falconiformes, and Strigiformes orders [21]. Corvids, which belong to the Passeriformes, are important reservoir hosts. Northern goshawks, which are members of Accipitridae but traditionally classified as Falconiformes, have been identified as highly susceptible to WNV infection. Consequent mortality of highly susceptible birds like Goshawks and Corvids acts as an indicator of active transmission domain, while *Cx* mosquitoes are the most important vector for WNV to date [21, 22]. Generally, goshawks, which are resident and short migratory birds, are an important indicator host for WNV in Europe [23]. This has been supported by records of dead wild birds in Germany, with two Northern goshawks affected in Saxony-Anhalt and Saxony in 2018 and 19 infected goshawks detected between 2018 and 2019 [8]. Birds of prey have also been identified as suitable indicators for the beginning of a wave of infection due to their high level of susceptibility to the virus, particularly Northern Goshawks [9]. Several studies have indicated that both migratory and residential birds play important roles in the introduction, circulation, and enzootic maintenance of WNV. Michel et al. [11] presented results from surveillance of birds across Germany, with high WNV antibodies being observed in residential and short-distance migratory birds, which were the first indicator of autochthonous WNV circulation in the country.

### State of the Art and Research Gaps

Previous modelling approaches have been attempted to explain WNV infection outbreak in Germany, but only at a limited spatial and temporal scale. Ziegler et al. [24] used extrinsic incubation period values to generate a spatial approximation of transmission risk for WNV across Germany. However, the approximated transmission risk could not be easily translated into terms of R_0_, which would help explain an epidemic outbreak. Bhowmick et al. [25] attempted to model WNV spread across Germany, but used temperature data from only two weather stations, leading to spatially-limited and temporally coarse results. Bergsman et al. [26], Laperriere et al. [27], and Pu et al. [28] developed compartmental models for WNV transmission at a coarse spatial and temporal resolution, using only temperature data as the sole climatic forcing by assuming that all adult mosquitoes go host-seeking and transmit pathogens.

Although several models can establish statistical relationships between environmental covariates, mosquito abundance and diseases outbreak, results may fall short of depicting a robust spatiotemporal trend, reducing reliability. This is because vector occurrence and abundance does not necessarily indicate or translate to an equal proportion of vector competence and vector capacity which is often reported in correlative based vector population model. Conflicting results due to environmental novelty at different spatial and temporal scales have been reported in several WNV risk models [29]. Nevertheless, knowledge gaps still exist in the spatiotemporal variation of transmission intensity, frequency, and seasonality.

With findings from surveillance of birds for WNV, variance in vector competence across mosquito taxa, and non-monotonic WNV transmission risk dynamics, an ideal approach would be a spatiotemporally explicit model that considers bird population dynamics and migration patterns, mosquito occurrence and abundance, as well as the influence of underlying environmental factors that affect vector distribution and disease transmission rates in Germany. A process-based mechanistic model calibrated with climatic data and functional traits of vector mosquitoes and host birds would be ideal support public health preparedness to address the spread of mosquito-borne diseases.

Here, we present a process-based mechanistic epidemic model, which was able to explicitly describe the complete vector population and the spatiotemporal dynamics of past WNV outbreak in Germany. We considered the distinction between mosquito population compartments that allowed only older host-seeking mosquitoes progress to the epidemic compartments to avoid homogeneous mixing. We also considered the role of migratory and residential birds in amplifying WNV cases across Germany. Functional traits of both categories of birds and mosquitoes were used for model calibration. We also derived spatiotemporally explicit R_0_ rates across Germany, which we compared to observed WNV occurrences.

## Materials and Methods

### Study Area

The study area encompassed the whole of Germany. Since 2018, when the first autochthonous WNV cases were recorded, it has become endemic and circulates among birds, equids, and humans [17]. The heat wave in 2018 across Europe, followed by an unusually wet spring, created excellent conditions for vectors to thrive and increase transmission risk due to a shortened gonotrophic cycle and decreased extrinsic incubation period [22]. To understand and project the spatiotemporal dynamics of WNV, we collected epidemiological and environmental data covering Germany, which were them used to calibrate, parameterize, and drive our spatiotemporally explicit epidemic model.

### Epidemiological, Environmental and Bird Data

**Fig. 1:**
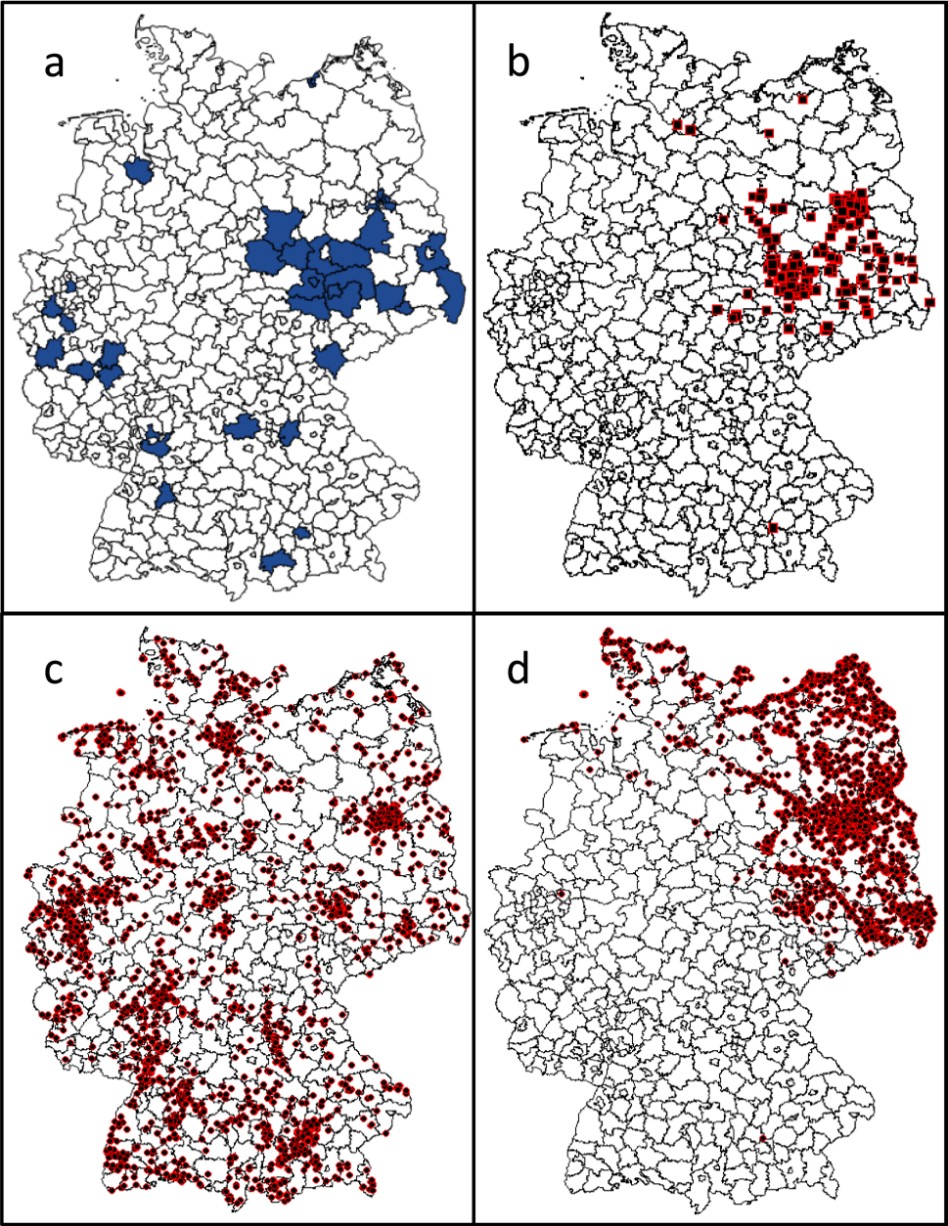
WNV cases in humans (**a**) and animals (**b**) across Germany between 2018 and 2022. The blue-coloured polygons in **a** are Nomenclature of Territorial Units for Statistics NUTS3 administrative areas where human WNV infections have been detected obtained from SurvStat database of Robert Koch-Institute. The black dots in **b** are locations where WNV infection has been detected in animals obtained from Animal Diseases Information System (TSIS) of Friedrich Loeffler Institute.

Since the first autochthonous cases in Germany, WNV transmission and occurrence, which includes cases in humans, birds, and equids from surveillance activities, has been well documented. For this research, active and resolved WNV cases were collected from the Animal Diseases Information System database of the FLI from 2018 to 2022 [30]. The database includes cases with detailed date and time stamps, location of occurrence, host type, and habitat type. Information on human cases, which included week, location, and year of occurrence, was collected from the Robert Koch Institute database [31] (Fig. 1).

Climate data is crucial to understand the dynamics of arboviral disease outbreaks. Introduction, circulation, and maintenance of an infectious vector-borne disease have been linked to changing climate. Air temperature, precipitation, and relative humidity were used as the key climatic forcings to replicate the fundamental niche responsible for supporting mosquito population and infection transmission risks in the study area. High resolution daily climate data for Germany was obtained from E-OBS between 2017 and 2022 [32].

Corvids have been identified as highly susceptible to WNV infection and Northern Goshawks as highly susceptible to death from infection [23]. The spatiotemporal occurrence data of migratory and residential birds between 2017 to 2022 were obtained from E-bird online database [33].

### Spatiotemporally Explicit WNV Epidemic Model

The fundamental processes that drive vector-borne diseases to spread in a population are complex. Understanding the biological mechanisms behind disease dynamics can aid in modelling disease spread. This includes identifying attributes required for a disease vector to attain its fundamental niche. Given that the entomology, life traits, and functional ecology of WNV vector and hosts are well understood, we adopted a mechanistic approach to design an epidemic model that explicitly predicts WNV outbreaks in Germany across space and time.

**Fig. 2:**
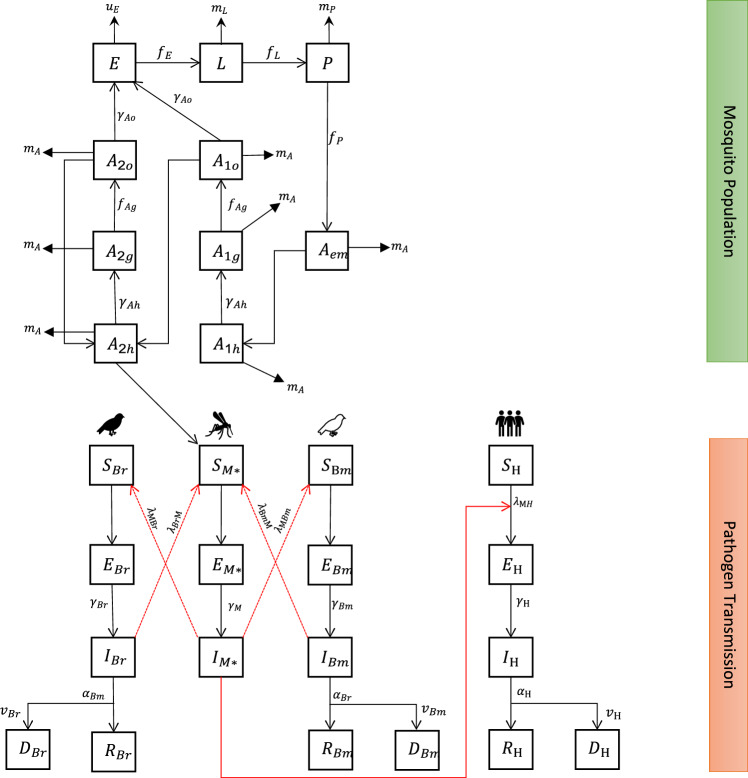
Diagram of epidemic model for WNV showing two sections depicting mosquito population and WNV infection explaining pathogen transmission between mosquito, resident birds, migratory birds, and humans. Eggs ($$\mathrm{E}$$) laid by adult mosquitoes develop to larvae (L), pupa (P), and then emerge as new adults ($${\mathrm{A}}_{\mathrm{em}}$$). A fraction of new adults go host seeking ($${\mathrm{A}}_{1\mathrm{h}}$$) after mating, rest ($${\mathrm{A}}_{1\mathrm{g}}$$), or oviposition ($${\mathrm{A}}_{1\mathrm{o}})$$ to lay new eggs. Older mosquitoes also go host-seeking ($${\mathrm{A}}_{2\mathrm{h}}$$), rest ($${\mathrm{A}}_{2\mathrm{g}}$$), and oviposition ($${\mathrm{A}}_{2\mathrm{o}}$$). Only host-seeking mosquitoes transit into the infection transmission compartment of the model to become susceptible mosquitoes ($${\mathrm{S}}_{\mathrm{M}})$$, which feed on residential birds ($${\mathrm{S}}_{\mathrm{Br}})$$ and migratory birds $$\left({\mathrm{S}}_{\mathrm{Bm}}\right)$$ and either infects exposed birds ($${\mathrm{E}}_{\mathrm{Br}},{\mathrm{E}}_{\mathrm{Bm}})$$ or becomes exposed ($${\mathrm{E}}_{\mathrm{M}})$$ after being infected ($${\mathrm{I}}_{\mathrm{M}})$$ by the birds. Infected birds are removed ($${\mathrm{R}}_{\mathrm{Br}},{\mathrm{R}}_{\mathrm{Bm}})$$ by either recovery or death ($${\mathrm{D}}_{\mathrm{Br}},{\mathrm{D}}_{\mathrm{Bm}}).$$ Susceptible humans ($${\mathrm{S}}_{\mathrm{H}})$$ become exposed $${(\mathrm{E}}_{\mathrm{H}})$$ when fed on by infected mosquitoes and may become infected and removed either by recovery $${(\mathrm{R}}_{\mathrm{H}})$$ or death $$({\mathrm{D}}_{\mathrm{H}})$$. Compartments with asterisk are modulated by adult mortality ($${m}_{A})$$

The model was developed using concepts from population biology and mathematical epidemiology. We applied a novel approach that considers vector population as well as disease transmission between vector and two different bird compartments. With this approach, we aimed to achieve reduced homogenous mixing during transmission and cross-infection. Adapted from Laperriere et al. [27], the compartment structure consists of two sections: one describing mosquito population growth and the other describing various health states and disease transmission between mosquitoes, residential birds, and populations of migratory birds (Fig. 2).

Mosquito and bird population were explained by a density dependent population growth model. This is because the population of both vector and hosts are modulated by seasonality and removal rates. This approach solves the issue that may arise in a classical exponential population growth rate, where seasonal trend in population growth is absent. The mechanistic model is dependent on frequency of contact between vector and amplifying host. It is also modulated by the density of both vector and host respectively.

### Vector and Host Population Dynamics

We developed a density dependent population growth model for mosquitoes forced by seasonal variation in temperature, precipitation, and relative humidity. Also, growth rates for migratory and residential birds were defined as a function of their natural birth and mortality rate.

The population growth for *Cx pipiens* mosquitoes was described by a ten-compartment ordinary differential equation (ODE) adapted from Tran et al. [34] and Ezanno et al. [35] in Eq. (1). Three aquatic stages and seven terrestrial stages were clearly accounted for, including birth and mortality rates. The aquatic stages were egg, larvae, and pupae, while the terrestrial stage was explained due to their behavior during the gonotrophic cycle, which is either host seeking, gravid, or ovipositioning [34]. Since infections are transmitted when mosquitoes feed on a host, only older mosquitoes from the host-seeking compartment ($\dot{{A}_{2h}}$) transited to the bird compartments to avoid the effect of homogenous mixing seen in previous studies by Bergsman et al. [26], Bhowmick et al. [25], Laperriere et al. [27], Pu et al. [28]; Rubel et al. [36], where mosquitoes were introduced as susceptible with no special compartment dedicated to explaining their life cycle. A disease-free mosquito population dynamic was explained by the following ODE:

1 $$\left\{\begin{array}{l}\dot{E}=d{\gamma }_{Ao}\left({\beta }_{1}{A}_{1o}+{\beta }_{2}{A}_{2o}\right)-\left({\mu }_{E}+d{f}_{E}\right)E\\ \dot{L}=d{f}_{E}E-\left({m}_{L}\left(1+L/{k}_{Lv}\right)+{f}_{L}\right)L\\ \dot{P}={f}_{L}L-\left({m}_{P}+{f}_{P}\right)P\\ \dot{{A}_{em}}={f}_{P}P\sigma e\left(-{\mu }_{em}\left(1+P/{k}_{Pv}\right)\right)-\left({m}_{A}+{\gamma }_{Aem}\right){A}_{em}\\ {\dot{A}}_{1h}={\gamma }_{Aem}\dot{{A}_{1h}}-\left({m}_{A}+{\mu }_{r}+{\gamma }_{Ah}\right){A}_{1h}\\ \dot{{A}_{1g}}={\gamma }_{Ah}{A}_{1h}-\left({m}_{A}+{\mu }_{pr}+{f}_{Ag}\right){A}_{1g}\\ \dot{{A}_{1o}}={f}_{Ag}{A}_{1g}-\left({m}_{A}+{\mu }_{r}+{f}_{Ao}\right){A}_{1o}\\ \dot{{A}_{2h}}={f}_{Ao}\left({A}_{1o}+{A}_{2o}\right)-\left({m}_{A}+{\mu }_{r}+{\gamma }_{Ah}\right){A}_{2h}\\ \dot{{A}_{2g}}={\gamma }_{Ah}{A}_{2h}-\left({m}_{A}+{\mu }_{pr}+{f}_{Ag}\right){A}_{2g}\\ \dot{{A}_{2o}}={f}_{Ag}{A}_{2g}-\left({m}_{A}+{\mu }_{r}+{f}_{Ao}\right){A}_{2o}\end{array}\right.$$

The process of infection and disease transmission begins when disease-free emergent adult mosquitoes (${A}_{em}$) go host-seeking for blood meal ($\dot{{A}_{1h}}$ and $\dot{{A}_{2h}}$) and feed on birds at a biting rate ($k$) driven by temperature. Although both nulliparous and parous mosquitoes go host-seeking and may become infected, only parous mosquitoes ($\dot{{A}_{2h}}$) can infect a bird through a blood meal given that the extrinsic incubation period of WNV in mosquitoes is longer than the gonotrophic cycle of mosquitoes. Hence, younger mosquitoes are unable to effect transmission. Some of the infected birds would have developed viremia enabling them to facilitate transmission to mosquitoes. Some mosquitoes become infected and keep infecting exposed birds that they feed on. This process of infection between mosquito and bird population is explained in Eq. (2). Although there has been evidence of vertical transmission from adult to egg, this is often a small proportion of infection as about 75% of infection would be lost from positive families during larval to adult development stage and was therefore not accounted for [37].

2 $$\begin{array}{c}\frac{\mathrm{d}{S}_{\mathrm{M}}}{\mathrm{d}t}=(-{\lambda }_{BrM}(T)+{\lambda }_{BmM}(T)){S}_{\mathrm{M}}{+A}_{2h}-{m}_{A}(T){S}_{\mathrm{M}}\\ \frac{\mathrm{d}{E}_{\mathrm{M}}}{\mathrm{d}t}={(\lambda }_{BrM}\left(T\right)+{\lambda }_{BmM}(T)){S}_{\mathrm{M}}-{\gamma }_{\mathrm{M}}(T){E}_{\mathrm{M}}-{m}_{A}(T){E}_{\mathrm{M}}\\ \frac{\mathrm{d}{I}_{\mathrm{M}}}{\mathrm{d}t}={\gamma }_{\mathrm{M}}(T){E}_{\mathrm{M}}-{m}_{A}(T){I}_{\mathrm{M}}\end{array}$$

The spatiotemporally explicit epidemic model includes dynamic pathogen transmission within the population of residential, migratory birds, and humans respectively. It is defined by the following ODE:

3 $$\begin{array}{l}\frac{\mathrm{d}{S}_{\mathrm{Br}}}{\mathrm{d}t}=\left({b}_{\mathrm{Br}}-\left({b}_{\mathrm{Br}}-{m}_{\mathrm{Br}}\right)\frac{{N}_{\mathrm{Br}}}{{K}_{\mathrm{Br}}}\right){N}_{\mathrm{Br}}-{\lambda }_{MBr}(T){S}_{\mathrm{Br}}-{m}_{\mathrm{Br}}{S}_{\mathrm{Br}}\\ \frac{\mathrm{d}{E}_{\mathrm{Br}}}{\mathrm{d}t}={\lambda }_{MBr}(T){S}_{\mathrm{Br}}-{\gamma }_{\mathrm{Br}}{E}_{\mathrm{Br}}-{m}_{\mathrm{Br}}{E}_{\mathrm{Br}}\\ \frac{\mathrm{d}{I}_{\mathrm{Br}}}{\mathrm{d}t}={\gamma }_{\mathrm{Br}}{E}_{\mathrm{Br}}-{\alpha }_{\mathrm{Br}}{I}_{\mathrm{Br}}-{m}_{\mathrm{Br}}{I}_{\mathrm{Br}}\\ \frac{\mathrm{d}{R}_{\mathrm{Br}}}{\mathrm{d}t}=\left(1-{v}_{\mathrm{Br}}\right){\alpha }_{\mathrm{Br}}{I}_{\mathrm{Br}}-{m}_{\mathrm{Br}}{R}_{\mathrm{Br}}\\ \frac{\mathrm{d}{D}_{\mathrm{Br}}}{\mathrm{d}t}={v}_{\mathrm{Br}}{\alpha }_{\mathrm{Br}}{I}_{\mathrm{Br}}\end{array}$$

4 $$\begin{array}{l}\frac{\mathrm{d}{S}_{\mathrm{Bm}}}{\mathrm{d}t}=\left({b}_{\mathrm{Bm}}-\left({b}_{\mathrm{Bm}}-{m}_{\mathrm{Bm}}\right)\frac{{N}_{\mathrm{Bm}}}{{K}_{\mathrm{Bm}}}\right){N}_{\mathrm{Bm}}-{\lambda }_{MBm}(T){S}_{\mathrm{Bm}}-{m}_{\mathrm{Bm}}{S}_{\mathrm{Bm}}\\ \frac{\mathrm{d}{E}_{\mathrm{Bm}}}{\mathrm{d}t}={\lambda }_{MBm}(T){S}_{\mathrm{Bm}}-{\gamma }_{\mathrm{Bm}}{E}_{\mathrm{Bm}}-{m}_{\mathrm{Bm}}{E}_{\mathrm{Bm}}\\ \frac{\mathrm{d}{I}_{\mathrm{Bm}}}{\mathrm{d}t}={\gamma }_{\mathrm{Bm}}{E}_{\mathrm{Bm}}-{\alpha }_{\mathrm{Bm}}{I}_{\mathrm{Bm}}-{m}_{\mathrm{Bm}}{I}_{\mathrm{Bm}}\\ \frac{\mathrm{d}{R}_{\mathrm{Bm}}}{\mathrm{d}t}=\left(1-{v}_{\mathrm{Bm}}\right){\alpha }_{\mathrm{Bm}}{I}_{\mathrm{Bm}}-{m}_{\mathrm{Bm}}{R}_{\mathrm{Bm}}\\ \frac{\mathrm{d}{D}_{\mathrm{Bm}}}{\mathrm{d}t}={v}_{\mathrm{Bm}}{\alpha }_{\mathrm{Bm}}{I}_{\mathrm{Bm}}\end{array}$$

5 $$\begin{array}{l}\frac{\mathrm{d}{S}_{\mathrm{H}}}{\mathrm{d}t}={r}_{\mathrm{H}}{N}_{\mathrm{H}}-{\lambda }_{MH}(T){S}_{\mathrm{H}}-{m}_{\mathrm{H}}{S}_{\mathrm{H}}\\ \frac{\mathrm{d}{E}_{\mathrm{H}}}{\mathrm{d}t}={\lambda }_{MH}(T){S}_{\mathrm{H}}-{\gamma }_{\mathrm{H}}{E}_{H}-{m}_{\mathrm{H}}{E}_{\mathrm{H}}\\ \frac{\mathrm{d}{I}_{\mathrm{H}}}{\mathrm{d}t}={\gamma }_{\mathrm{H}}{E}_{\mathrm{H}}-{\alpha }_{\mathrm{H}}{I}_{\mathrm{H}}-{m}_{\mathrm{H}}{I}_{\mathrm{H}}\\ \frac{\mathrm{d}{R}_{\mathrm{H}}}{\mathrm{d}t}=\left(1-{v}_{\mathrm{H}}\right){\alpha }_{\mathrm{H}}{I}_{\mathrm{H}}-{m}_{\mathrm{H}}{R}_{\mathrm{H}}\\ \frac{\mathrm{d}{D}_{\mathrm{H}}}{\mathrm{d}t}={v}_{\mathrm{H}}{\alpha }_{\mathrm{H}}{I}_{\mathrm{H}}\end{array}$$

Since we considered only the adult stage of birds, one compartment of ODE was sufficient to explain the logistic growth of both migratory and residential bird populations driven by natural birth, mortality rate, and carrying capacity adapted from [27, 36].

### Infection and Cross-Infection

Infection and cross infection between populations are crucial to maintain the natural enzootic cycle of WNV. Feeding pattern and host preference of WNV vectors are essential to stimulating infection and cross infection between vector and host population. *Cx pipiens* mosquitoes play a crucial role in maintaining WNV natural transmission cycle. Also, *Cx pipiens* biotype hybrids are also important vectors for spillover of WNV to humans due to their preference to feed on mammals [16]. Although *Cx torrentium* has been identified as a mosquito with high vector competence for WNV, they seem to occur at similar locations with *Cx pipiens* and were accounted for by the mosquito population model.

A temperature dependent cross infection regime was defined by a temperature and density dependent process to describe the process of transmission and force of infection between mosquitoes to residential birds, mosquitoes to migratory birds, residential birds to mosquitoes, and migratory birds to mosquitoes, respectively. As described by Laperriere et al. [27], it is a frequency dependent process following a similar process for malaria transmission. $\dot{{A}_{1h}}$ $\dot{{A}_{2h}}$.

6 $${\lambda }_{MBr}(T)={\delta }_{M}{F}_{b}k(T){p}_{MBr}{\phi }_{Br}\frac{{I}_{M}}{{k}_{Mv}}$$

7 $${\lambda }_{MBm}(T)={\delta }_{M}{F}_{b}k(T){p}_{MBm}{\phi }_{Bm}\frac{{I}_{M}}{{k}_{Mv}}$$

8 $${\lambda }_{MH}(T)={\delta }_{M}{F}_{h}k(T){p}_{MH}{\phi }_{Bm}\frac{{I}_{M}}{{k}_{Mv}}$$

9 $$\begin{array}{c}{\lambda }_{BrM}(T)={\delta }_{M}{F}_{b}k(T){p}_{BrM}\frac{{I}_{Br}}{{K}_{Br}}\\ \end{array}$$

10 $${\lambda }_{BmM}(T)={\delta }_{M}{F}_{b}k(T){p}_{BmM}\frac{{I}_{Bm}}{{K}_{Bm}}$$

The process of transmission and cross infection between mosquitoes and the residential and migratory birds were described in Eqs. (6) and (7) as a product of host preference of mosquito ($${F}_{b})$$, ($${F}_{h})$$ observed for *Cx*. *pipiens* mosquitoes in Germanys, biting rate ($$k$$) of mosquitoes on birds, portion of active mosquitoes ($${\delta }_{M}$$), probability of infection transmission from mosquitoes to birds ($${p}_{MBr,}{p}_{MBm})$$, ratio of mosquito to birds on both categories ($${\phi }_{Br},{\phi }_{Bm})$$, portion of infected mosquitoes ($${I}_{M})$$ and carrying capacity of mosquitoes in the region ($${K}_{M})$$ [27, 38]. Similarly, Eqs. (9) and (10) describe the process of transmission and cross infection between both bird categories and mosquitoes. The mosquito to host ratio ($${\phi }_{Br},{\phi }_{Bm}),$$ which averages the number of bites that a host receives per unit time, is an important factor during the cross infection and disease transmission process, given that our model is density and contact frequency dependent [27]. Also, the portion of active mosquitos is key to defining the trend in biting regime. For humans, which are dead end hosts, cross-infection is absent and is only defined by a population density and contact rate function in Eq. (8).

### Parameter Estimation and Model Calibration

Epidemic model parametrization is crucial to attaining a stable and reliable description that explains the outbreak being studied. For this research, we carefully studied and compared several parameters previously used in similar studies to select the best fit. Some were assumed with the closest relationship to reality while others were sourced from functional traits of vectors and hosts included in the study (see Supplementary Material for *Initial Bird Parameters*). Parameters of interest for residential and migratory birds, which includes mortality rate, as well as infection and removal rates, were carefully estimated from similar studies and documented (Table 1). Parameters for humans were adopted from [27] (Table 2).

**Table 1:** Parameters for birds

Parameter	Description	Value	Source
${\gamma }_{\mathrm{Br}}$	Infectious rate residential bird	0.196	[24]
${\gamma }_{\mathrm{Bm}}$	Infectious rate migratory bird	0.285	[24]
${\alpha }_{\mathrm{Br}}$	Removal rate residential bird	0.867	[24]
${\alpha }_{\mathrm{Bm}}$	Removal rate migratory bird	0.4	Fitted
${m}_{\mathrm{Br}}$	Natural death rate residential bird	0.0005	[60]
${m}_{\mathrm{Bm}}$	Natural death rate migratory bird	0.00023	[61]
${v}_{\mathrm{Br}}$	Death rate due to infection for residential bird	0.655	[24]
${v}_{\mathrm{Bm}}$	Death Rate due to infection for migratory bird	0.103	[24]

Per unit capita rates are in unit days

Birth rate for birds (${\mathrm{B}}_{\mathrm{br}}$, ${\mathrm{B}}_{\mathrm{bm}}$) were estimated from their observed monthly individual juvinile abundance [62]

**Table 2:** Parameters for humans

Parameter	Description	Value	Source
${b}_{\mathrm{H}}$	Birth rate humans	0.000055	[27]
${\gamma }_{\mathrm{H}}$	Infectious rate humans	0.25	[27]
${\alpha }_{\mathrm{H}}$	Removal rate humans	0.5	[27]
${m}_{\mathrm{H}}$	Natural death rate humans	0.000034	[27]
${v}_{\mathrm{H}}$	Death rate due to infection for humans	0.004	[27]

Per unit capita rates are in unit days

The population of mosquitoes was driven by climatic variables. Parameters used as input to describe seasonal mosquito growth were obtained from laboratory validated functional traits of *Cx pipiens* mosquitoes from published literature. These parameters were either dependent or independent of climatic factors and have been documented (see Supplementary Material Table S1 and S2).

Certain parameters used as inputs in process-based models can be estimated from a distribution or range of values, which can introduce uncertainties in model structure and output. For our model, temperature-independent parameters were estimated from a certain range of values, hence the need for sensitivity analysis and inverse calibration for improved parameter optimization. The method applied here has been documented (Supplementary Material for *Model Calibration and Parameter Optimization* and Table S3), (Table 3).

**Table 3:** Temperature independent transmission determinants

Parameter	Description	Value	Source
${p}_{MBr}$	Probability of transmission from mosquito to residential bird	0.97	[27]
${p}_{MBm}$	Probability of transmission from mosquito to migratory bird	0.9	[27]
${p}_{MH}$	Probability of transmission from mosquito to humans	0.5	Fitted
${p}_{BrM}$	Probability of transmission from residential bird to mosquito	0.4	Fitted
${p}_{BmM}$	Probability of transmission from migratory bird to mosquito	0.7	Fitted
${F}_{b}$	Proportion of mosquito feeding on birds	0.25	[38]
${F}_{h}$	Proportion of mosquito feeding on humans	0.38	[38]

Per unit capita rates are in unit days

Fitted values were estimated using inverse calibration method

### Transmission Functions and Basic Reproductive Number

Some important transmission parameters described as functions are determinants of contact rate, pathogen replication, and force of infection. They are driven by temperature, vector to host ratio, and daylight length (Table 4). The biting rate of mosquitoes ($\kappa$) was determined by their gonotrophic cycle, which is driven by temperature. Extrinsic incubation period (${\gamma }_{M}),$ which describes the effective pathogen replication rate was also driven by temperature. The percentage of non-hibernating mosquitoes (${\delta }_{M}$) was defined by a function of daylight length.

**Table 4:** Transmission determinants defined by functions

Parameter	Function	Source
$\kappa (T)$	$\frac{0.344}{1+1.231\mathrm{e}(-0.184(\mathrm{T}-20))}$	[27]
${\gamma }_{M}\left(T\right)$	$0.0093T-0.1352$ if *T*>15, else 0	[27]
${\phi }_{Br}$	$\frac{{S}_{\mathrm{Br}}}{{k}_{Mv}(T)}$	Fitted
${\phi }_{Bm}$	$\frac{{S}_{\mathrm{Bm}}}{{k}_{Mv}(T)}$	Fitted
${\phi }_{Bm}$	$\frac{{S}_{H}}{{k}_{Mv}(T)}$	Fitted
${\delta }_{M}$	$1-\frac{1}{1+1775.7\mathrm{exp}\left[1.559\left(D-18.177\right)\right]}$ $D=7.639arcsin\left[tan\left(\epsilon \right)tan\left(\varphi \right)+\frac{0.0146}{cos\left(\epsilon \right)cos\left(\varphi \right)}\right]+12$ $\epsilon =0.409sin\left(\frac{2\pi \left(d-80\right)}{365}\right)$	[38]
		[38]

Per unit capita rates are in unit days

Basic reproductive number also known as R_0_ is one of the most important results expected from the model. It represents the number of secondary infections that will occur from the introduction of a single infectious vector to a susceptible population, where baseline conditions are met. It was computed based on the next-generation matrix approach described by Diekmann et al. [39] using the ODE below:

11 $$R_{0} = \sqrt {\left( {\frac{{\gamma_{{\mathrm{M}}} \left( T \right)\beta_{Mbr} \left( T \right) + \beta_{Mbm} \left( T \right)}}{{\left( {\gamma_{{\mathrm{M}}} \left( T \right) + m_{{\mathrm{M}}} \left( T \right)} \right)m_{{\mathrm{M}}} \left( T \right)}} \frac{{S_{{{\mathrm{Br}}}} }}{{K_{{{\mathrm{Br}}}} }} + \frac{{S_{{{\mathrm{Bm}}}} }}{{K_{{{\mathrm{Bm}}}} }}} \right)\left( {\left[ {\frac{{\gamma_{{{\mathrm{Br}}}} \beta_{{{\mathrm{Br}}}} \left( T \right)}}{{\left( {\gamma_{{{\mathrm{Br}}}} + m_{{{\mathrm{Br}}}} } \right)\left( {\alpha_{{{\mathrm{Br}}}} + m_{{{\mathrm{Br}}}} } \right)}}\frac{{S_{{{\mathrm{M}}}} }}{{K_{{{\mathrm{Br}}}} }}} \right] + \left[ {\frac{{\gamma_{{{\mathrm{Bm}}}} \beta_{{{\mathrm{Bm}}}} \left( T \right)}}{{\left( {\gamma_{{{\mathrm{Bm}}}} + m_{{{\mathrm{Bm}}}} } \right)\left( {\alpha_{{{\mathrm{Bm}}}} + m_{{{\mathrm{Bm}}}} } \right)}}\frac{{S_{{{\mathrm{M}}}} }}{{K_{{{\mathrm{Bm}}}} }}} \right]} \right)}$$

### Application and Model Simulation

Population structure and disease states described in (Fig. 2) were explicitly simulated to obtain outputs that were verified and validated using occurrence data. Simulation of WNV epidemic dynamics began in January and ended in December between 2017 and 2020. Initial values of all compartments were set to 0, with the exception of mosquito eggs (E), infected mosquitoes (${I}_{\mathrm{M}})$, susceptible residential birds (${S}_{\mathrm{Br}})$, susceptible migratory birds (${S}_{\mathrm{Bm}})$, and susceptible humans (${S}_{\mathrm{H}})$. Unlike previous studies, such as the approach used by Laperriere et al. [27, 30, minimum number of adult mosquitoes was not constant, but a factor of mosquito population dynamics in a natural cycle. Furthermore, only older host seeking mosquitoes ${(A}_{2h})$ transited into the disease transmission section of the model, making this approach novel.

Carrying capacity for mosquitoes were of two kinds. The first was the standard carrying capacity, assuming an optimal environmental condition using an approach by Kerkow et al. [40]. Secondly, a modulated carrying capacity was computed from the standard carrying capacity, modified by the trend of relative humidity. A novel approach was used to introduce a spatially explicit carrying capacity for both resident and migratory birds. Spatial abundance data for hooded crow and Northern goshawk were obtained from bird occurrence data available in bird online databases, which are recorded at the NUT3 level between 2017 and 2020 [33].

Mosquito to host ratio, which is a key transmission parameter, was then computed using the estimated number of susceptible mosquitoes ${(S}_{m}$), number of residential birds $\left({K}_{Br}\right)$, and susceptible migratory birds (${K}_{Br}$). Unlike previous studies, which used the ratio obtained from the total number of mosquitoes, we used the daily number of susceptible mosquitoes ${(S}_{m}$) at each location for our computation.

**Fig. 3:**
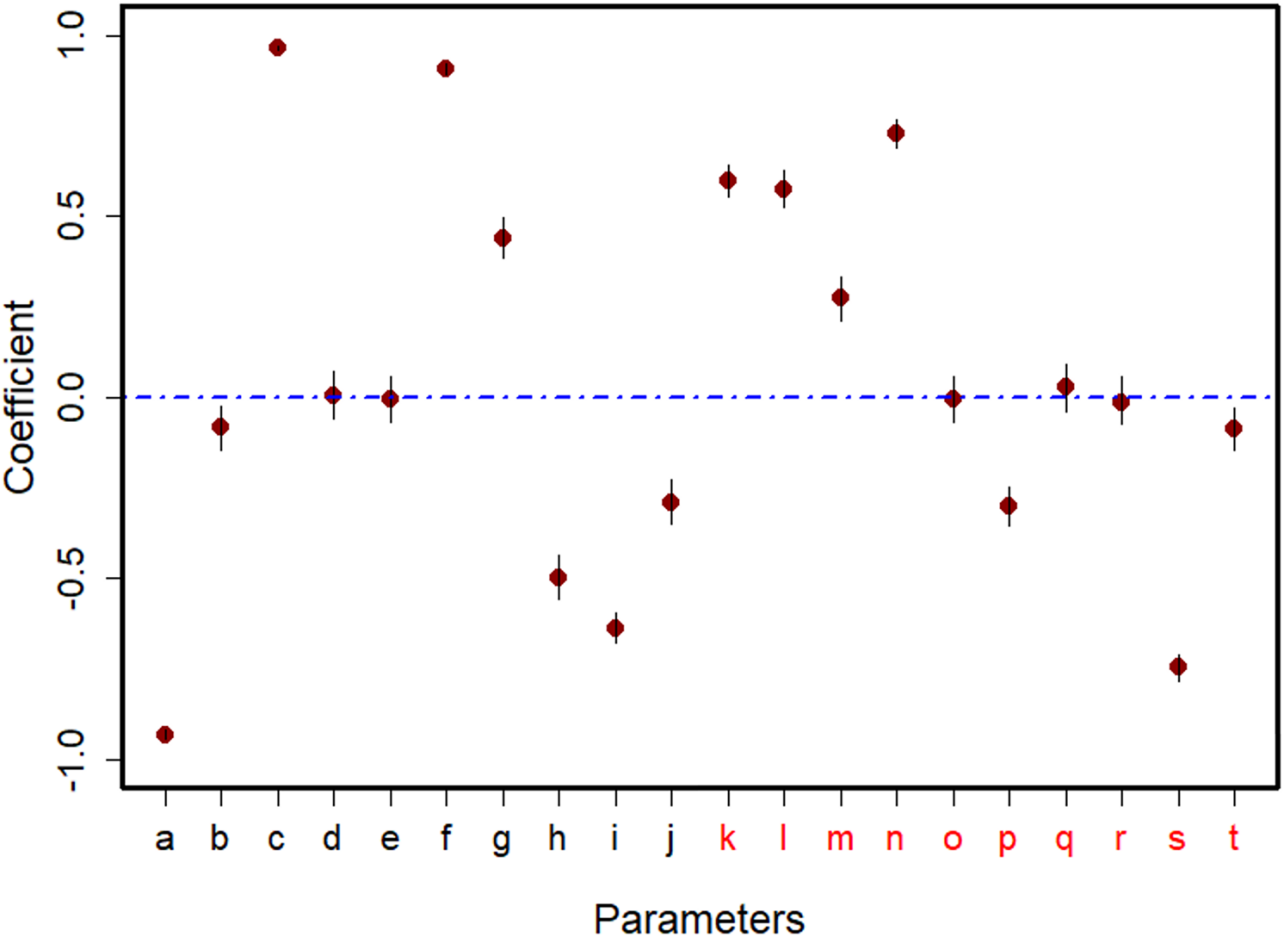
Sensitivity analysis using the Latin Hypercube Sampling approach and Partial Rank Correlation Coefficient showing the magnitude and direction of each parameter on model output. The dashed blue lines indicate a near zero effect on our selected output of interest (R_0_). This step is needed to identify parameters for inverse calibration using Bayesian inference method. Mosquito population parameters are labelled in black while pathogen transmission parameters are labelled in red. For the rates, ***a*** egg mortality, ***b*** minimum pupae mortality, ***c*** sex ratio at emergence, ***d*** minimum adult mortality, ***e***minimum lavae mortality, ***f*** oviposition rate, ***g*** adult development rate, ***h*** host-seeking rate, ***i*** risky behavior mortality, ***j*** mortality during emergence, ***k*** mosquito to residential bird transmission probability, ***l*** mosquito to migratory bird transmission probability, ***m*** residential bird to mosquito transmission probability, ***n***migratory bird to mosquito transmission probability, ***o*** infectious rate in resident bird, ***p*** removal rate of residential bird, ***q*** death rate of residential bird due to infection, ***r*** infectious rate in migratory bird, ***s*** removal rate of migratory bird, ***t***death rate of migratory bird due to infection

Inverse calibration approach, which entails estimating appropriate values from selected parameter ranges, was used to optimize estimated parameter selection during our model calibration. (See Supplementary Material Table S3). For this, we used the Bayesian estimation method. It utilizes the bayes theorem approach which requires a likelihood, prior, and evidence to determine a posterior. Daily occurrence of WNV cases obtained from FLI Animal Diseases Information System [30] were used to derive our likelihood, while our priors were selected from a range of possibilities for each parameter to improve model reliability both spatially and temporally. Sensitivity analysis was carried out to identify parameters that affect R_0_ outputs. The parameters with the most influence were used to execute inverse calibration to select a final parameter value with the highest posterior probability density (Fig. 3) [41]. Implementation of the model was done numerically in R programming language Version 4.3.0 [42].

## Results

### Mosquito Population and Pathogen Transmission Dynamics

**Fig. 4:**
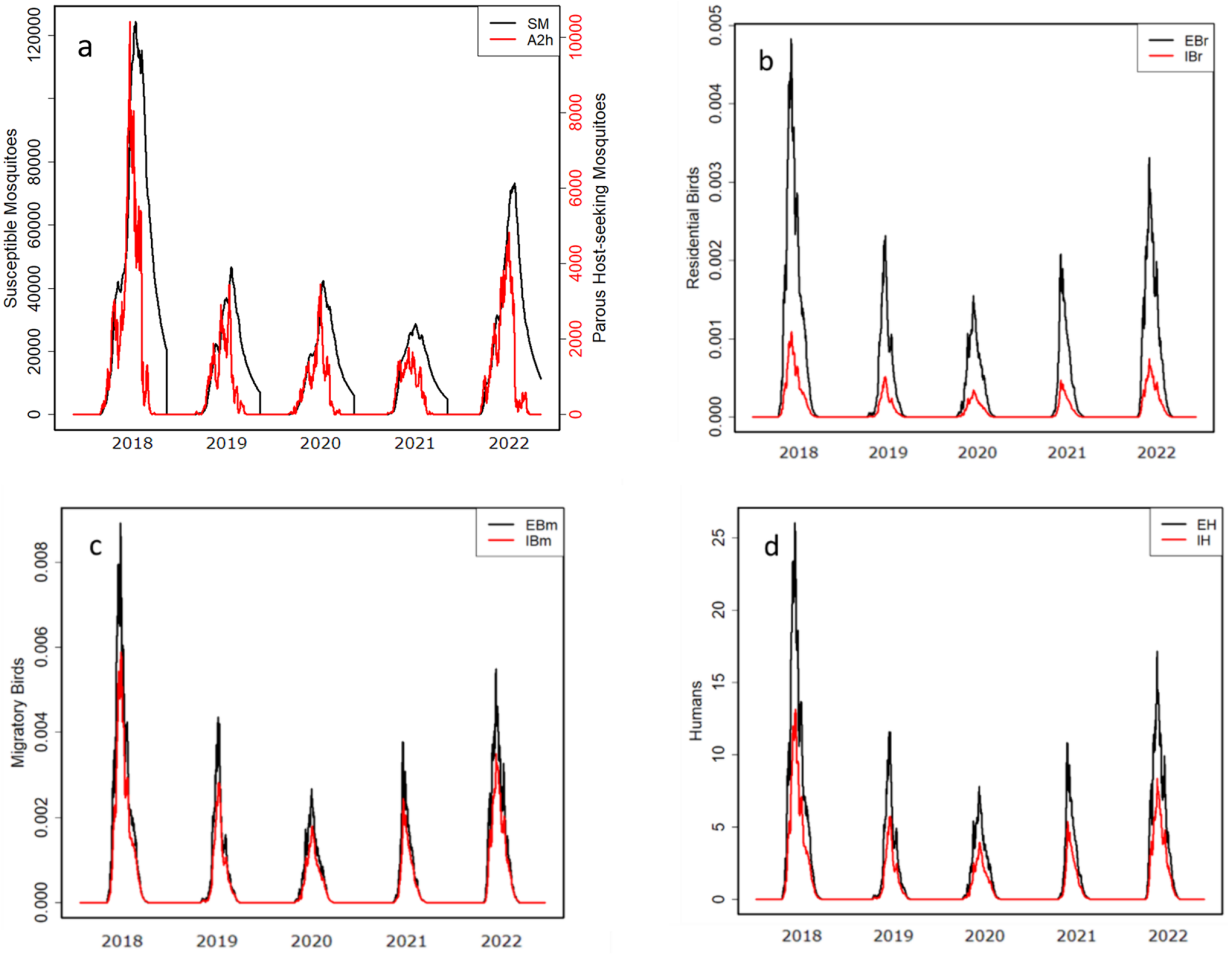
Simulated time series of dynamics of population and health states of mosquitoes (**a**), residential birds (**b**), migratory birds (**c**) and humans (**d**) between 2018 and 2022

Abundance of mosquitoes, actively driven by climatic variables, was simulated by our epidemic model with a clear seasonal pattern. Our model was able to simulate a seasonal dynamic of pathogen transmission between compartments at different stages for mosquitoes, birds, and humans (Fig. 4) Infection in residential and migratory birds were driven by the health state of mosquito population, with both exhibiting a similar trend (Fig. 4).

### Thermal Response of Transition Rates and Infection Traits

Thermal responses generated by our model were consistent with *Cx pipens* trait responses. Fecundity, mosquito development rates, mortality rates, biting rate, and extrinsic incubation rate of WNV all responded to temperature variation. Fecundity was lowest at about 6 °C, peaked at 25 °C, and further reduced with an increase in temperature. Pupae to adult development was activated at about 5 °C, but was very low until about 10 °C. Mortality rates of adult mosquitos increased with temperatures below 25 °C. For biting rate, we observed that host-feeding activities increased linearly with temperature from 5 °C until about 30 °C. Extrinsic incubation rate was activated at an average temperature slightly above 20 °C and terminated at temperatures slightly below 30 °C. These key conditions were crucial in explaining the dynamics of vector population and infection transmission across space and time. Graphs for thermal responses have been documented (See Supplementary Material Fig S1.).

### WNV Infection Trend across Germany

**Fig. 5:**
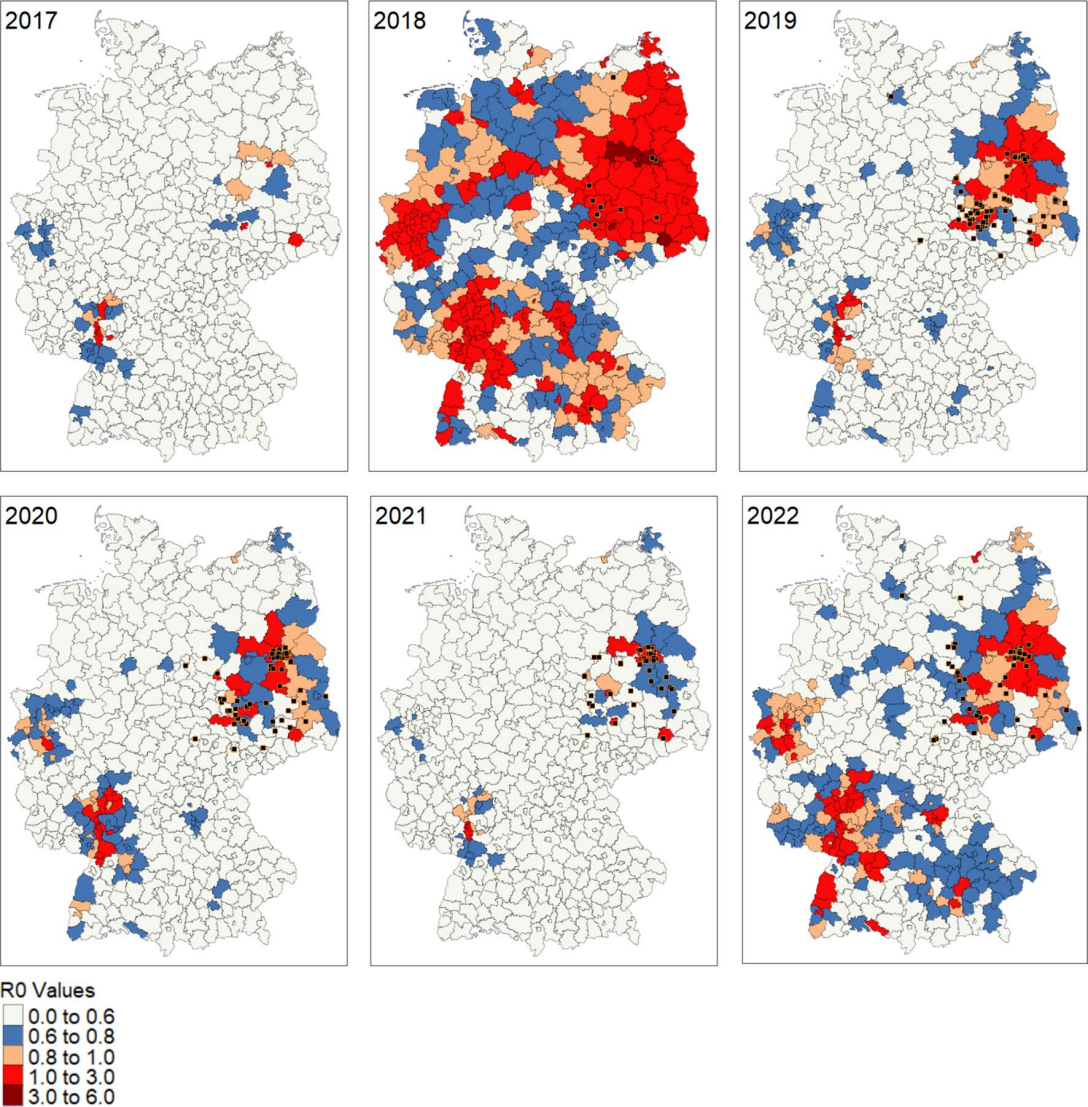
R_0_ values across Germany at NUT3 level from 2017 to 2022. Maps were estimated from daily R_0_ values averaged between August and October (week 30–42) when the peak of the WNV infections was detected. The black squares represent high resolution WNV occurrence records in animals obtained from Animal Diseases Information System (TSIS) database of FLI. No occurrence was recorded in 2017

Spatiotemporally explicit daily output of R_0_, depicting number of active infections at the NUTS 3 administrative level across Germany between 2017 and 2022, reveals four major hotspots of possible WNV transmission: East Germany, Western North Rhine-Westphalia, upper and middle Rhine areas and individual NUTS3 regions in Bavaria (Fig. 5). This was a product of mosquito population dynamics and disease transmission regime, with spatial differences corresponding to spatial variation of climatic forcings that drive mosquito transition rates and disease transmission between model compartments. The number of days where R_0_ exceeded 1.0 varied across years, with 41 days in 2017 with no observed outbreak, 114 days in 2018 with the first cases observed, 77 days in 2019, 57 days in 2020, 45 days in 2021, and 86 days in 2022.

### Seasonality of WNV Infection

**Fig. 6:**
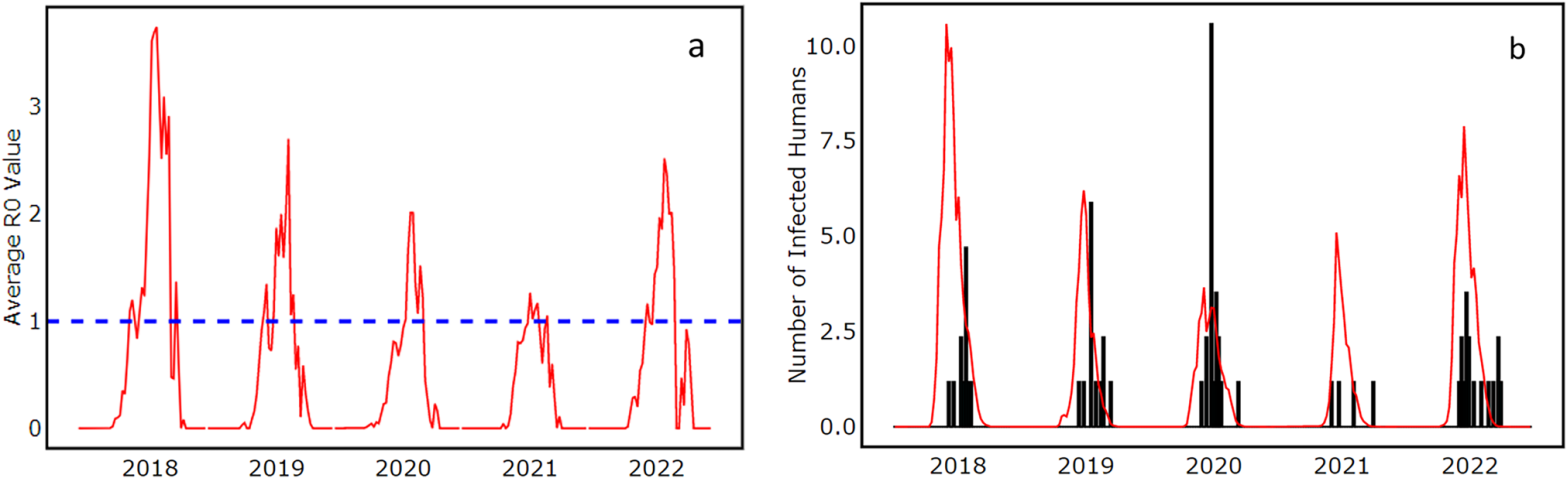
Infection transmission rates for animals expressed as R_0_ values across Germany from 2018 to 2022 are shown in **a** The red line represent simulated daily R_0_ values aggregated weekly. The blue dashed horizontal line is the threshold where infection transmission becomes active equivalent to R_0_ rates above 1.0. The number of infected humans across Germany from 2018 to 2022 is shown in **b**. The red lines represent simulated daily numbers of infected humans aggregated weekly. The black vertical bars represent observed cases of WNV infection

**Fig. 7:**
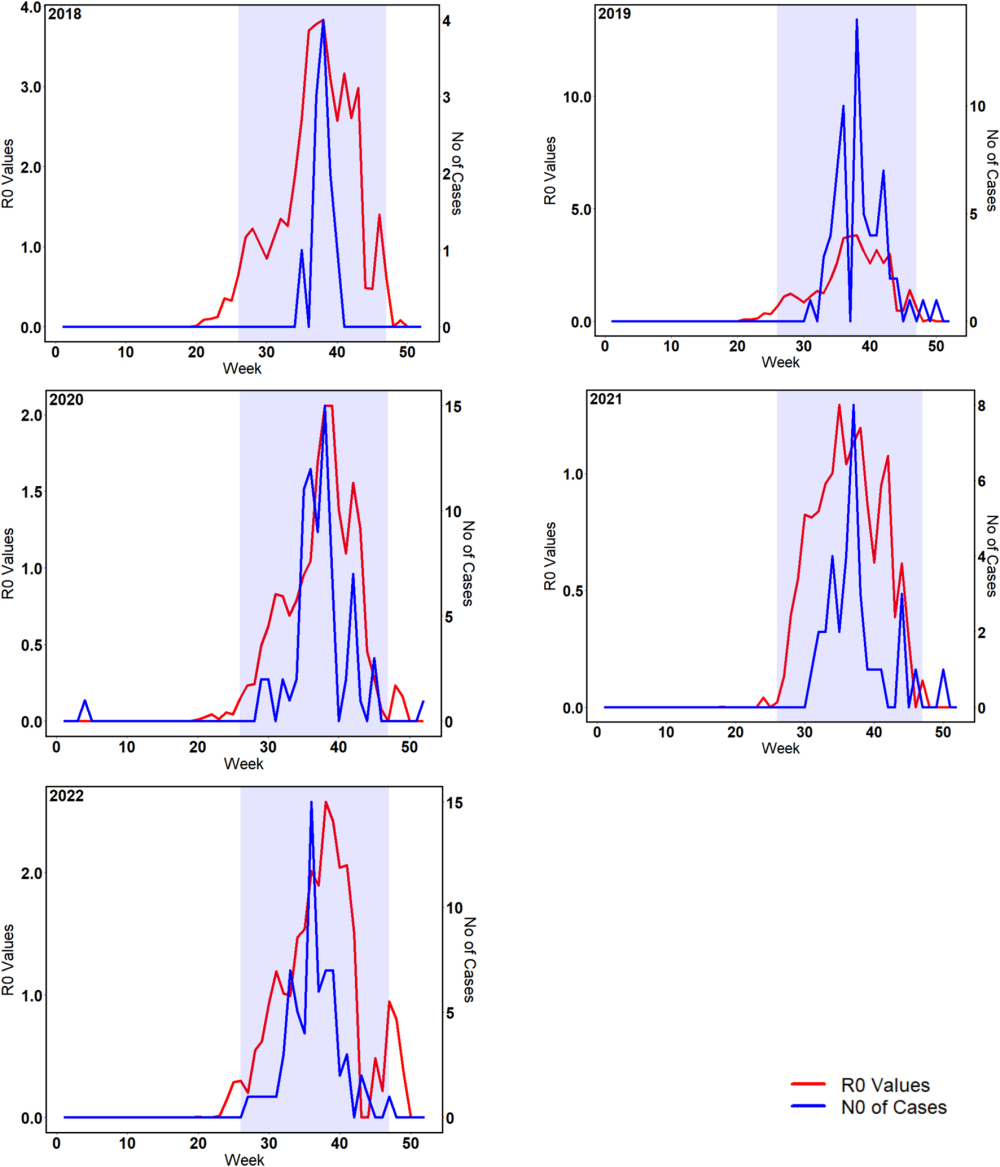
Weekly bird infection transmission rates expressed as R_0_ values and weekly animal cases of WNV infection across Germany from 2018 to 2022. The shaded rectangular area shows weeks when disease transmission is likely active, which is ideally two weeks after the beginning of mosquito season and two weeks after the end of mosquito season in Germany. This can be translated to begin at week 26 and end in week 47

Regardless of the multi-stage spatiotemporal mosquito abundance pattern, R_0_ rates that depict infection did not correlate with mosquito population abundance pattern. Instead, R_0_ rates were driven by a combination of several factors, such as vector population, vectoral capacity, and vector competence, with infection parameters such as biting rate of mosquitoes and extrinsic incubation rate playing key roles. This resonates with the role of environmental suitability in infectious disease transmission dynamics. Most of the observed WNV cases obtained from FLI and RKI in animals and humans occurred between week 27 and 45. Time lags of 5 and 10 weeks were applied to model simulated R0 rates and infected human cases respectively which has been applied in several mosquito borne disease models to account for time it takes for mosquito population to increase, seek host, acquire pathogen, become infectious, bite and infect a host; host develops symptoms, humans host seek treatment and report cases [63]. Our model results had a similar trend with the occurrences and R0 values crossing the threshold level of 1.0 between weeks 27 and 45 respectively (Fig. 7). Infected human cases simulated by our model also showed a similar pattern with observed human cases (Fig. 6). Our results (Fig. S1) validate the reports that *Cx.* mosquito populations found in Germany are highly susceptible to WNV at relatively low temperatures and will even effectively transmit the virus at temperatures as low as 18 °C [43].

### Model Validation

**Fig. 8:**
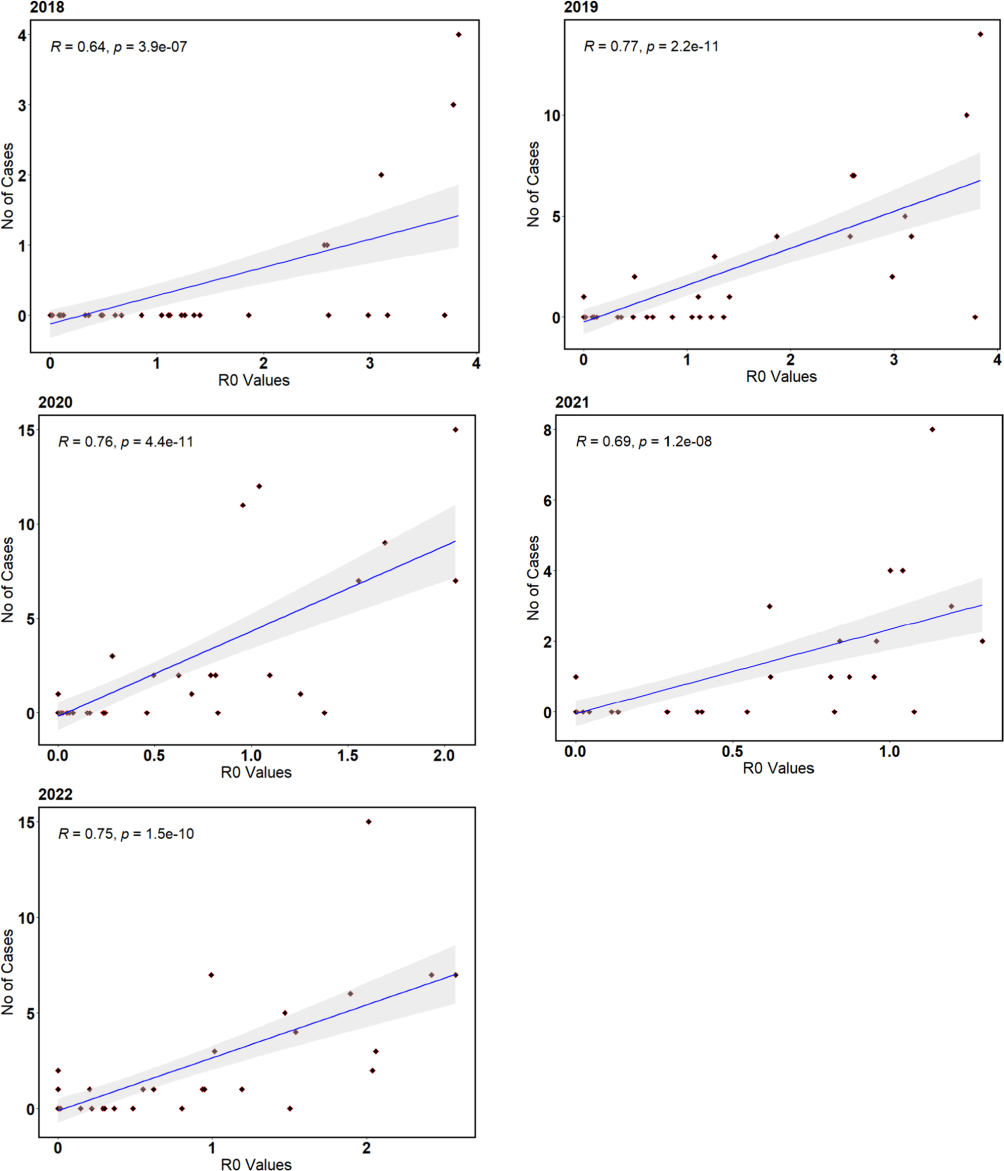
Observed number of cases and simulated R_0_ values for birds between 2018 and 2022, with *p* values and *R*^*2*^ values reported for each year at 95% confidence interval level. Black dots are weekly R_0_ values and observed number of cases while the blue line is the regression line with a light grey shaded confidence interval area

Statistical validation was used to ascertain model performance. Daily occurrence of WNV infections, which was initially used for parameter selection and model optimization, were compared to results obtained from model simulated R_0_ rates. Visually, the modelled R_0_ rates across Germany had similar spatial and temporal pattern to the occurrence records for animal and human cases (Figs. 5, 6, and 7). These findings were in line with our research question that an epidemic model forced with appropriate climatic variables and parameters would reproduce similar patterns with observed occurrences. Statistically, the strength of relationship between observed and simulated infection rates was tested using a linear regression with *R*^2^ and *p* values (Fig. 8). Simulated results for all periods had *R*^2^ values above 0.5 and *p* values below 0.005.

## Discussion

### Response of Functional Trait and WNV Infection to Climatic Forcings

Our model was able to simulate daily trends of mosquito population abundance and generate maps of infection transmission rates (R_0_ rates) across Germany from 2017 to 2022. Trends in climate variables are an important driver of mosquito-borne diseases and critical to understanding dynamics of vector occurrence, abundance, pathogen development, and disease transmission. On this premise, we developed a spatially and temporally explicit epidemic model for WNV in Germany. Our model was able to replicate WNV occurrences as R_0_ rates with spatial and temporal similarity across Germany on a fine scale, with Baden-Württemberg, Bavaria, Berlin, Brandenburg, Hamburg, North Rhine-Westphalia, Rhineland-Palatinate, Saarland, Saxony-Anhalt, and Saxony identified as states with elevated risk level. Interestingly, these states all recorded cases of WNV in humans, animals, or both between 2019 and 2022.

In 2018, an unprecedented climate anomaly was recorded across Europe, with temperatures higher than the 1981–2010 average, rainfall of more than 200 percent above the 1971–2000 average in countries like Greece which clearly supported early mosquito activities and resulted to counties recording early outbreaks and spontaneous increase in cases of WNV infections [7, 44, 45].Germany also had an unusual dry year and warmest since 1881 recorded the first cases that same year [43].

Functional traits that drove mosquito populations responded strongly to thermal trends in a unimodal form, with clearly defined minimum, optimum, and maximum temperatures [46, 47]. Fecundity and pupae to adult development rates all peaked between 23 and 25 °C, although fecundity was still possible after 25 °C, but reduced afterwards. These results were in agreement with [46]. This implies that the number of new mosquitoes reduced with increases in temperature across geographical space and time. Adult mosquito lifespan, which is the inverse of mosquito development rate, reduced with an increase in temperature, which could result in a reduced survival rate in areas with higher temperatures [47]. This is interesting and suggests that with the upward trend in temperature anomaly, *Cx pipiens* mosquito-borne diseases might likely reduce in areas with extremely high temperatures that reduce the lifespan of *Cx pipiens* mosquitoes.

Transmission parameters also responded to thermal trends in a unimodal form, varying across space time due to spatial and temporal heterogeneity in climate data used to force our model [47, 48]. Mosquitoes were actively host-feeding at temperatures as low as 10 °C, clearly showing a distinct behavior of European *Cx pipiens* population slightly different from reported biting rate from previous studies on temperature suitability of WNV establishment in Europe [49]. However, extrinsic incubation rate, which defines the rate of pathogen replication in a vector, was not active until 15 °C [50]. Both traits peaked at about 25 °C and reduced as temperature increased further. These responses were evident from the pattern observed in WNV infection occurrences simulated by our model as R_0_ rates across Germany. Infection was frequent two weeks after the beginning of mosquito season in temperate regions, which usually begins early June and lasts till the end of September or mid-October [51, 52]. The difference between the beginning of mosquito season and WNV infection outbreak was clearly a result of the incubation period needed for WNV pathogen to develop in a mosquito before it can be transmitted to a host, with *Cx* mosquitoes having an average of 10–14 days incubation period. Clinical symptoms generally become obvious between 2 and 14 days in humans and birds and 2–9 days in horses after being fed on by an infected mosquito [53]. Cases of WNV infection occurrences were reduced and subsequently absent for periods with little or no mosquito activities, which agreed with our simulated R_0_ rates and infected humans, which was below 1.0 and most times 0 for these periods (Figs. 5, 6, 7). Seasonal trends observed from our results were similar to trends reported across temperate Europe, exhibiting similar climate patterns as in Germany, with the majority of the infections occurring between June and October, since the beginning of the outbreak in 2018 [7, 9, 24, 54].

### Role of Migratory Bird in Sustaining WNV Enzootic Cycle

R_0_ rates from model simulation produced a similar spatial and temporal pattern to WNV and migratory bird occurrence data, but not with residential bird occurrence (Fig. 1). This could be explained by the significance of the spatial distribution of the migratory birds (hooded crow) predominantly found in Eastern Germany [33]. It is also interesting to note that the hooded crow and the American crow, which were the most reported bird mortalities from WNV infection in a study by Laperriere et al. [27], both belong to the *Corvus* family. Hooded crow has also been identified as an important short-distance migratory bird that supports maintenance of WNV enzootic cycle. From our results, it is obvious that they are susceptible to WNV infection and are likely to develop viremia and support transmission, but with reduced mortality compared to Northern goshawks. The choice of habitat for residential birds (northern goshawks) are coniferous forests, with over than 60% canopy-closure and this will ideally translate to Pines from the genus *Pinus* [55]. However, northern goshawks could also be found in any forest-type during winter, which coincidentally is a period when mosquitoes are inactive and with no active infection transmission. According to data of dominant tree species in Germany by Blickensdörfer et al. [56], pines are predominantly found in Eastern Germany, which is also a hotspot for WNV occurrence. Although bird data from E-bird online database shows that Northern Goshawk occurs in several parts of Germany, it is obvious that the environmental condition required for introduction and maintenance of WNV natural cycle is only attained Eastern Germany and a small part of the Rhine valley.

Colonization of urban areas by hooded crow, especially in European cities, can be traced to forest fragmentation, availability of food in urban areas, and competition for habitat space in rural areas with other birds [57]. These birds move to urban areas and help with the introduction and spill-over of pathogens like WNV in cities and sub-urban settlements. With food and habitat availability in urban and sub-urban areas, their migration is reduced, making them active as a primary host for WNV in cities like Berlin, which had record numbers of WNV infection cases. Although not occurring in Eastern Germany, the carrion crow, which is the closest relative to the hooded crow and the American crow, has shown to develop high rates of viremia and death when inoculated with WNV [58]. Findings from our research did not only show the susceptibility of hooded crow, but also revealed the potential of carrion crow, which occurs in Western Germany, more as a potential primary and amplifying host of WNV in Germany and Europe.

### Effect of Increased Number of Climatic Forcings, Aquatic Stage Inclusion and Homogenous Mix Control on Model Output

The novelty of our approach was hinged upon several concepts, either introduced or modified to achieve a more realistic and reproducible model output. The introduction of other climatic forcings that modulate environmental conditions determines dynamics of vector occurrence, abundance, and disease transmission rates. Relative humidity was able to effectively modulate the spatiotemporal carrying capacity of mosquitoes in urban and rural settlements. Rainfall modulated the oviposition rate of mosquitoes across Germany. This will also be key to reproducing historical and future trends of infectious diseases and is an essential approach to identify key drivers to be used in setting up early warning systems.

We successfully introduced a complete stage-structured mosquito population section to our model, which also included the aquatic stages usually exempted in previous studies to avoid model complexity and reduce computation time. One of the major problems solved by this approach was the elimination of possible saturation that assumes every adult mosquito translates to a susceptible mosquito compartment, including male mosquitoes, female gonotrophic mosquitoes in resting stages, and ovipositioning mosquitoes.

Only the older host-seeking mosquitoes that have lived long enough to support pathogen replication transited to become susceptible mosquitoes and feed on hosts including birds, humans, and animals enabled disease transmission by infection and cross infection. This approach eliminated homogenous mixing, which assumes that all female mosquitoes facilitate disease transmission at all times and places when active, which is not the case.

## Conclusion

Our model was able to reproduce spatially and temporally explicit R_0_ maps showing dynamics of WNV occurrences across Germany, which was associated with the deviation from daily means of climatic forcings. Currently, WNV is endemic in Eastern Germany due to the availability of suitable environmental conditions. Our results also show that transmission is likely to continue spreading towards other parts of Germany, potentially in parts of Baden-Württemberg. It was obvious that WNV infection was driven by climate dynamics. Functional traits of mosquitoes and transmission parameters were driven by climate forcings, especially temperature. Seasonality of WNV infection was described by our model, with simulated R_0_ rates similar to WNV infection cases recorded between 2018 and 2022. Infection predominantly occurred between week 27 and week 45, with the exception of 2018, where an early infection period was observed due to the heat wave across Europe. From our research, it was observed that short distance migratory birds were very important in the WNV infection transmission cycle. We suggest that Hooded crow, which occurred predominantly around Eastern Germany, is an important bird for maintenance of WNV transmission cycle in Germany. They can also develop viremia, become infected, support cross infection to mosquitoes, and persist in the population due to low mortality from infection. Although Northern goshawk (residential bird) is widely distributed across Germany, their impact on transmission and enzootic cycle maintenance for WNV was limited due to high mortality after infection. They instead serve as good indicators for new wave of WNV infection.

With reference to the index criteria listed by de Wit et al. [59], our study deviated from the Ross-Macdonald to consider certain improvements. Mosquito population was explicitly modelled from the aquatic to adult stages with multiple compartments. We also considered two bird taxa, one identified as the residential bird and the other as the migratory bird. Important functional traits and transmission rates such as mosquito development, biting, mortality rates, and extrinsic incubation period were driven by climatic variables. Force of infection was a function of host-feeding preference, transmission probability, and mosquito to host ratio. Also, the spatial nature of climatic forcings used accounted for spatial heterogeneity of vectors. The spatial nature of bird observation data accounted for spatial heterogeneity of the host species. Nevertheless, certain limitation exists in our model due to data constraints. Our model did not account for more than one mosquito taxa. Waning immunity, co-infection, vertical transmission and vector control were not included in the model.

To improve results from similar research in future, increasing the number of bird compartments in the model to account for a more detailed transmission effect of various bird species and their corresponding differential in WNV transmission dynamics would be promising. Different mosquito taxa, including hybrids, can be considered to set up a different model with a similar approach. Additionally, designing models that could account for transmission effects of different strains of WNV could be helpful in managing outbreaks of severe WNV strains and monitoring possible evolutionary trends. Furthermore, introduction of other environmental variables such as land-use type, landscape fragmentation, and hydrological structure of the study area into the process-based models could further increase model accuracy.

## Supplementary Information

Below is the link to the electronic supplementary material.

Supplementary file1 (DOCX 141 KB)

## Data Availability

Data and materials used for this research are available on request.

## Abbreviations

NUTS: Nomenclature of Territorial Units for Statistics
R0: Basic reproductive number
RKI: Robert Koch Institute
FLI: Friedrich-Loeffler-Institute
WNV: West Nile Virus
ODE: Ordinary differential equation
