# Correction: Spatiotemporally Explicit Epidemic Model for West Nile Virus Outbreak in Germany: An Inversely Calibrated Approach

**DOI:** 10.1007/s44197-025-00498-4

**Published:** 2026-02-27

**Authors:** Oliver Chinonso Mbaoma, Stephanie Margarete Thomas, Carl Beierkuhnlein

**Affiliations:** 1 Department of Biogeography, University of Bayreuth, Universitaetsstr. 30, 95447 Bayreuth, Germany; 2 Bayreuth Center of Ecology and Environmental Research, BayCEER, University of Bayreuth, Universitaetsstr. 30, 95447 Bayreuth, Germany; 3 Geographical Institute of the University of Bayreuth, GIB, Universitaetsstr. 30, 95447 Bayreuth, Germany; 4 Departamento de Botánico, Universidad de Granada, Granada, 18071 Spain

**Correction: Journal of Epidemiology and Global Health (2024) 14:1052-1070**

10.1007/s44197-024-00254-0

In this article, in the caption to Fig. 1, the labels ‘a’ and ‘b’ were swapped. The caption was incorrectly given as

**Fig. 1** WNV cases in animals (**a**) and humans (**b**) across Germany between 2018 and 2022. The black dots in **a** are locations where WNV infection has been detected in animals obtained from Animal Diseases Information System (TSIS) of Friedrich Loeffler Institute. The blue-coloured polygons in **b** are Nomenclature of Territorial Units for Statistics NUTS3 administrative areas where human WNV infections have been detected obtained from SurvStat database of Robert Koch-Institute. **c** represents observations of Northern goshawk while **d** represents observations of Hooded crow across Germany between 2018 and 2022 respectively

and should have read

**Fig. 1** WNV cases in humans (**a**) and animals (**b**) across Germany between 2018 and 2022. The blue-coloured polygons in **a** are Nomenclature of Territorial Units for Statistics NUTS3 administrative areas where human WNV infections have been detected obtained from SurvStat database of Robert Koch-Institute. The black dots in **b** are locations where WNV infection has been detected in animals obtained from Animal Diseases Information System (TSIS) of Friedrich Loeffler Institute. **c** represents observations of Northern goshawk while **d** represents observations of Hooded crow across Germany between 2018 and 2022 respectively.

Furthermore, Eq. 8 was incorrectly given as $\:{\lambda\:}_{MH}\left(T\right)={\delta\:}_{M}{F}_{h}k\left(T\right){p}_{MH}{\varphi\:}_{Bm}\frac{{I}_{M}}{{K}_{H}}$ and should have been $\:{\lambda\:}_{MH}\left(T\right)={\delta\:}_{M}{F}_{h}k\left(T\right){p}_{MH}{\varphi\:}_{Bm}\frac{{I}_{M}}{kMv}$ .

Also, in Table 4 of this article, the second entry in the second column was incorrectly given as ‘0.0093T − 0.1352’ and should have been ‘0.0093T − 0.1352 if T > 15, else 0’.

The original article has been corrected.

