# Supplementary material for: Spatiotemporally Explicit Epidemic Model for West Nile Virus Outbreak in Germany: An Inversely Calibrated Approach
Source: J Epidemiol Glob Health. 2024 Jul 4;14(3):1052–70. doi: 10.1007/s44197-024-00254-0 (PMC11442818; doi:10.1007/s44197-024-00254-0)
Supplement: Supplementary file 1 — Supplementary file1 (DOCX 141 KB) [file 44197_2024_254_MOESM1_ESM.docx]

**Supplementary Information 1 Methods**

**Initial Bird Parameters**

Information obtained from literature search on bird surveillance in Germany aided the selection of our choice of residential bird species [1],[2]. Raptors have always appeared to be of interest in studies about WNV bird host in Germany and Europe. [3] reported during a bird surveillance only 4 survived out of 19 northern goshawks that tested positive for WNV. This is also in agreement with other data obtained from TSIS platform of FLI. Also in agreement with our choice are several studies across Europe about the role of raptors in WNV outbreak. [4] reported that raptors have high susceptibility to WNV which eventually leads to high mortality rate from the infection. They have an average life expectancy of 10.5 years. In a surveillance by [5], 26 birds were trapped, and radio tracked and monitored in the city of Oulu Finland, 7 birds died, which resulted to a mortality rate of 0.25 per year or 0.0005 per day. Report from a bird surveillance program by [2] revealed that in 2019, out of 18 northern goshawks tested, 5 were positive to WNV and 10 were positive out of 31 tested in 2020. These were the highest amongst all birds tested and translated to an infectious rate of 0.295. Mortality rate due to infection was also computed from a compilation from [3] that among a total of 19 northern goshawk that tested positive for WNV, only four survived, translating to a mortality rate due to infection of 0.79.

For migratory bird species, data obtained from TSIS platform of FLI revealed that Corvids (Hooded crow) were quite susceptible to WNV with several of them appearing frequently second to raptors and tits. Also, research on bird surveillance reported hooded crow which is a short distance migrant as the most susceptible bird of the Corvidae family, generally sported in Eastern Germany all year round [2]. The mortality rate of hooded crows was estimated from a study by [6]. from this study, it was observed that 39 deaths were recorded from a population of 686 in the first year of the experiment while 55 deaths were also recorded from a population of 419 birds. This will result in an annual average mortality rate of 0.085 and 0.00023 per day. [2] also reported that only 1 amongst 13 birds tested was positive 1n 2019 and 2 out of 32 birds tested in 2020. This translated to an infectious rate of about 0.19. Mortality due to infection was estimated to about 0.103. Removal rate was assumed to be the same with that of American Crows from [10] which was 0.4.

**Initial Mosquito Parameters**

***Climate Independent Parameters***

We adopted most of our climate independent parameter values from [7] and [8] for aquatic and terrestrial stages for *Cx pipiens* mosquitoes with the exception of environmental carrying capacity which was estimated from values suggested by [9]. Others were fitted using the Bayesian inference inverse calibration method.

**Table S1.** Climate independent parameters for mosquitoes. Per unit capita rates are in unit days. Parameters were estimated from literature with exception of mortality related to predators and standard carrying capacities estimated by calibration.

| **Parameter** | **Description** | **Value** | **Source** |
| --- | --- | --- | --- |
| $\sigma$ | Sex-ratio at emergence | 0.5 | [7] |
| $\gamma_{Aem}$ | Development rate of Emerging adult | 0.75 | [7] |
| $\gamma_{Ah}$ | Transition rate from host seeking to engorged adult(day−1) | 0.885 | [7] |
| $\gamma_{Ao}$ | Minimum transition rate from host seeking to engorged adult(day−1) | 0.33 | [7] |
| $\mu_{e}$ | Minimum egg mortality rate(day−1) | 0.0262 | [8] |
| $\mu_{l}$ | Minimum larvae mortality rate(day−1) | 0.0304 | [8] |
| $\mu_{p}$ | Minimum pupae mortality rate(day−1) | 0.0146 | [8] |
| $\mu_{A}$ | Minimum adult mortality rate (day-^1^) | 0.015 | [8] |
| $\mu_{em}$ | Mortality rate during emergence(day−1) | 0.1 | [7] |
| $\mu_{r}$ | Mortality rate related to seeking behavior (day−1) | 0.08 | [8] |
| $\mu_{pr}$ | Mortality rate related to predators (day−1) | 0.4 | Fitted |
| $TDD_{Ag}$ | Number of degree-days needed for egg maturation | 64.4 | [8] |
| $T_{Ag}$ | Minimal temperature needed for egg maturation | 9.8 | [8] |
| $k_{Lf}$ | Standard carrying capacity for larvae (per Km^2^) | 31,875 | [9] |
| $k_{Pf}$ | Standard carrying capacity for pupae | $k_{Lf}$*0.97 | Fitted |
| $k_{Mf}$ | Standard capacity for Mosquito | $k_{Pf}$*0.9 | Fitted |

**Climate Dependent Functions**

In process-based models, parameters driven by an external forcing are usually represented by a function. Climate dependent development rates were driven by temperature, precipitation, and relative humidity.

**Table S2.** Climate dependent parameters defined by functions. Per unit capita rates are in unit days.

| **Variable** | **Description** | **Function** | **Source** |
| --- | --- | --- | --- |
| $f_{E}$ | Transition rate from egg to larvae | $0.16\left( e^{0.105\left( T-10 \right)} \right)-e^{0.105\left( 35-10 \right)-(35-T)/5.007}$*(*Rhnorm*) | [8] |
| $f_{L}$ | Transition rate from larvae to pupae | $f_{p}$/4 | [8] |
| $f_{P}$ | Transition rate from pupae to emerging adults | $0.021\left( e^{0.162\left( T-10 \right)} \right)-e^{0.162\left( 35-10 \right)-(35-T)/5.007}$*1+*Pnorm* | [8] |
| $f_{Ag}$ | Transition from engorged to ovipositing adult | $\left( T-T_{\mathrm{Ag}} \right)/\mathrm{TD}D_{\mathrm{Ag}}\text{ }\text{ if }T>T_{Ag}$ | [8] |
| $f_{Ao}$ | Transition rate from ovipositing to host-seeking adults | $\gamma_{Ao}$*(1+*Pnorm*) | [7] |
| $m_{L}$ | Larvae mortality rate | $\mu_{L}+e^{-T/2}$ | [8] |
| $m_{P}$ | Pupae mortality rate | $\mu_{p}+e^{-T/2}$ | [8] |
| $m_{A}$ | Adult mortality rate | -0.05941+0.002965T | [8] |
| $k_{Lv}$ | Modulated carrying capacity larvae | *A**$k_{Lf}*Rh$ | Fitted |
| $k_{Pv}$ | Modulated carrying capacity pupae | *A**$k_{Pf}*Rh$ | Fitted |
| $k_{Mv}$ | Modulated carrying capacity Mosquito | *A**$k_{Mf}*Rh$ | Fitted |

*A* is the area of study location, *Pnorm* is normalised daily precipitation with ranges between 0 and 1, *Rhnorm* is normalised humidity with ranges between 0 and 1.

**Model Calibration and Parameter Optimization**

Certain parameters used as input in process-based models can be estimated from a distribution or range of values. This can introduce uncertainties in model structure and output. For our model climate independent parameters were estimated from certain range of values (initial parameters) hence the need for sensitivity analysis and subsequent calibration and parameter optimization. To identify parameters with the most influence on model output, quantify possible parameter errors and selection of valid range of values, we used sensitivity analysis implemented by the Partial rank correlation coefficient method to analyze the interaction between parameters and the effect of a single parameter to the most important output of our model, which was R0 rate in our case. We used Bayesian inference for inverse calibration which utilizes the bayes theorem approach by using the likelihood, prior, and evidence to determine a posterior estimated from the range of values obtained from literatures (Table S3). Parameters with known ranges extracted from literature were varied across those ranges while parameters with fixed values were varied by approximated range of values.

**Table S3.** Climate independent parameters used for sensitivity analysis and inverse calibration.

| **Parameter** | **Range** | **Calibrated Value** | **Source** |
| --- | --- | --- | --- |
| $\mu_{E}$ | 0.01-0.0262 | 0.015 | [8] |
| $\mu_{P}$ | 0.01-0.0146 | 0.01 | [7],[8] |
| $\sigma$ | 0.5 | 0.5 | [8] |
| $\mu_{A}$ | 0.01-0.023 | 0.015 | [8] |
| $\mu_{L}$ | 0.01-0.0304 | 0.03 | [7],[8] |
| $\gamma_{Ao}$ | 0.33-2 | 0.33 | [7],[8] |
| $\gamma_{Aem}$ | 0.5-0.75 | 0.54 | [7] |
| $\gamma_{Ah}$ | 0.33-0.885 | 0.549 | [7],[8] |
| $\mu_{r}$ | 0.05-0.08 | 0.072 | [8] |
| $\mu_{em}$ | 0.08-0.15 | 0.109 | [8] |
| $p_{MBr}$ | 0.7-1.0 | 0.9 | [10] |
| $p_{MBm}$ | 0.7-1.0 | 0.97 | [10] |
| $p_{BrM}$ | 0.3-0.7 | 0.4 | Fitted |
| $p_{BmM}$ | 0.3-0.7 | 0.7 | Fitted |
| $\gamma_{\mathrm{Br}}$ | 0.15-0.3 | 0.196 | [3] |
| $\alpha_{\mathrm{Br}}$ | 0.3-1 | 0.867 | [3] |
| $v_{\mathrm{Br}}$ | 0.6-0.8 | 0.655 | [3] |
| $\gamma_{\mathrm{Bm}}$ | 0.19-0.3 | 0.285 | [3] |
| $\alpha_{\mathrm{Bm}}$ | 0.1-0.5 | 0.40 | [10] |
| $v_{\mathrm{Bm}}$ | 0.1-0.2 | 0.103 | [3] |

**Supplementary Information 2 Results**

**Thermal Response of Transition Rates and Infection Traits.**

| 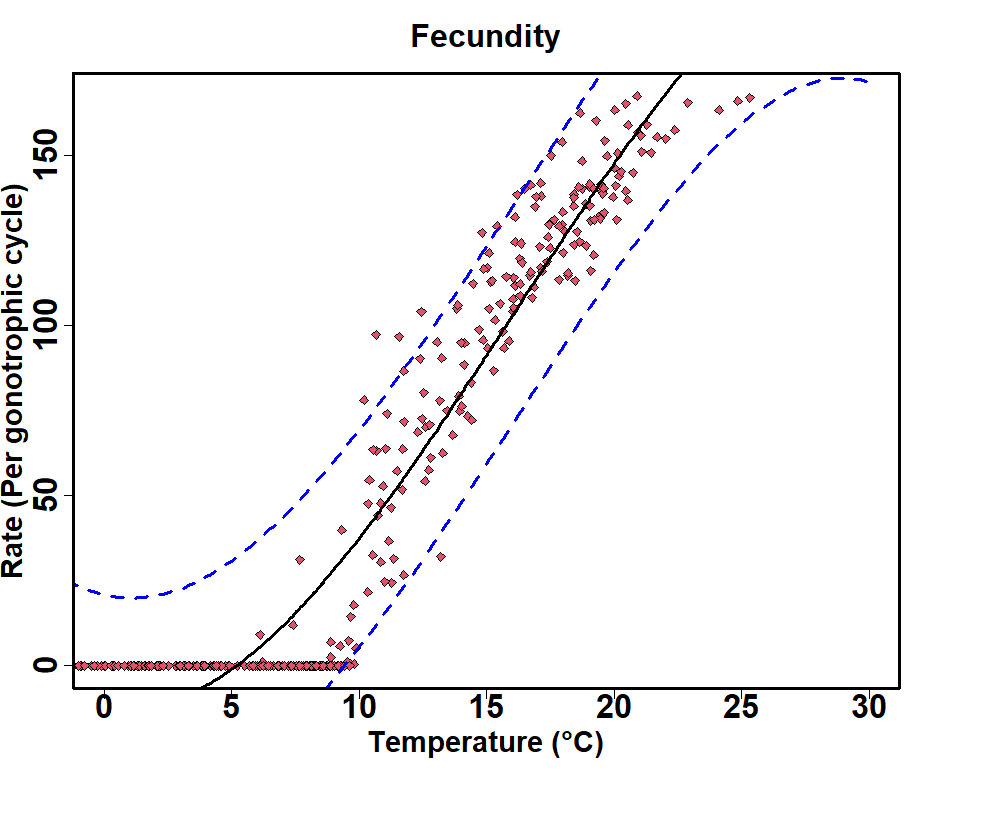 | 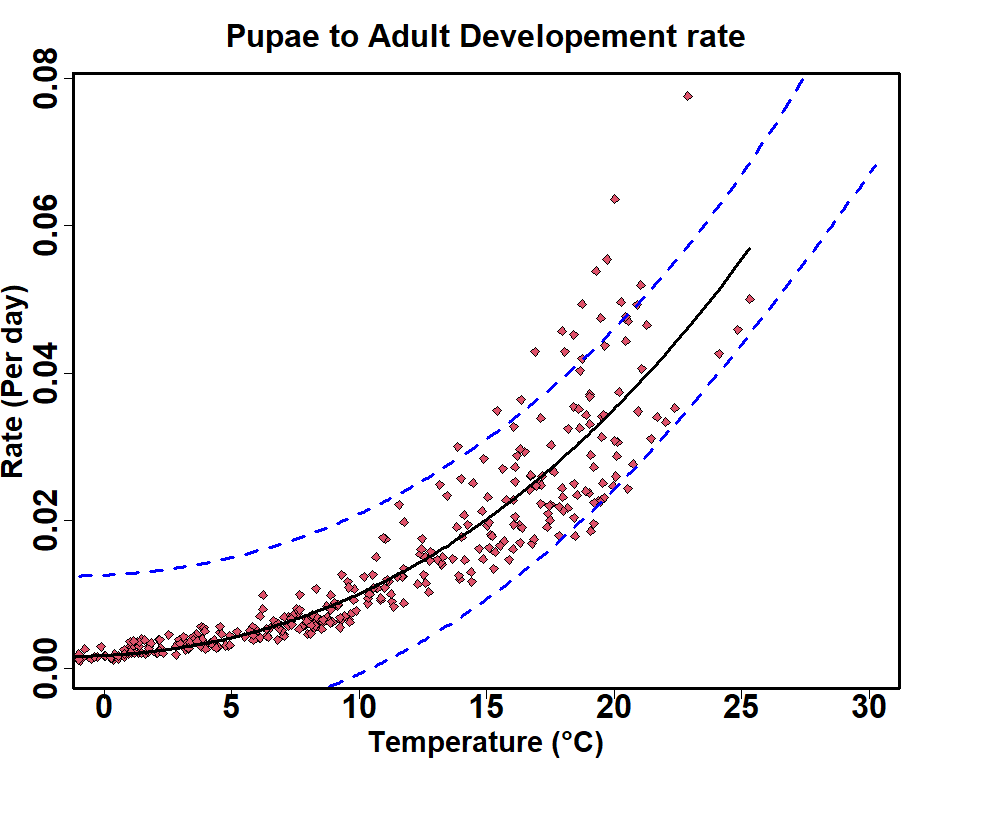 |
| --- | --- |
| 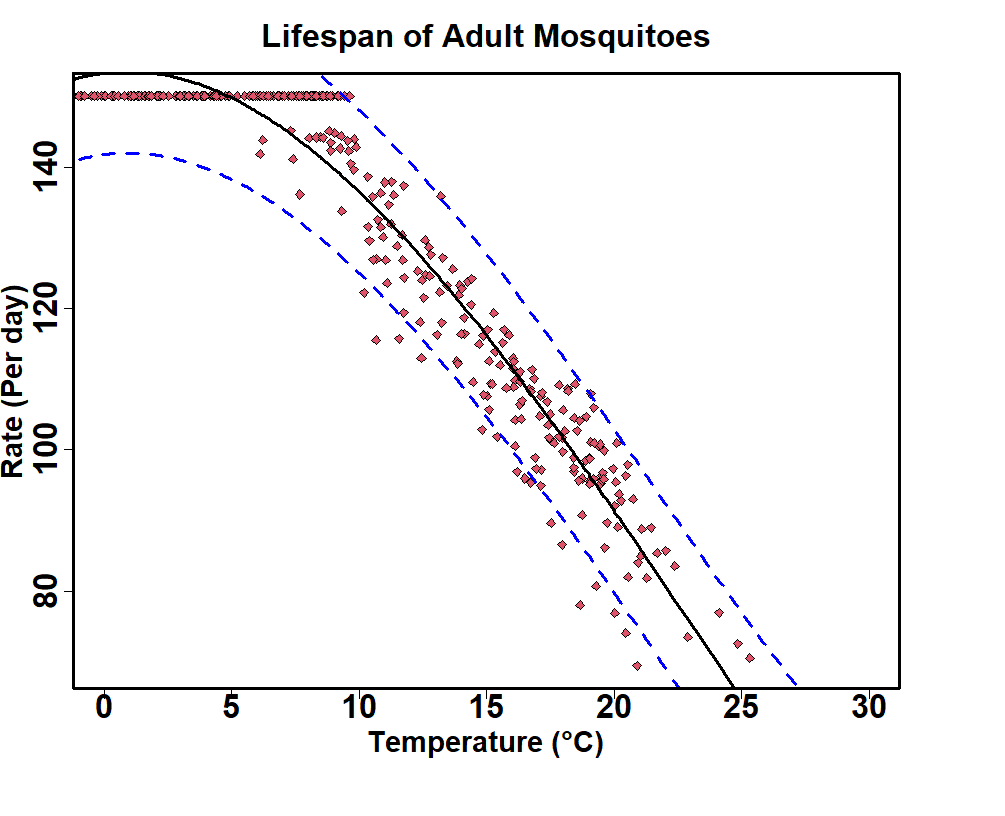 | 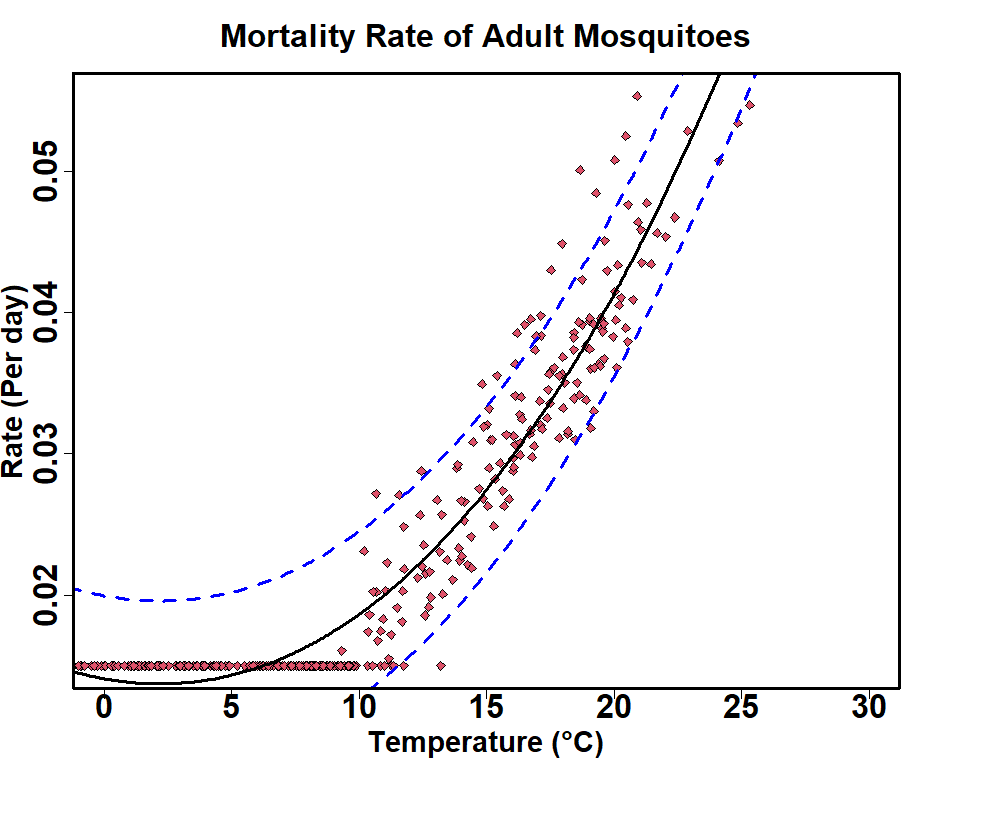 |
| 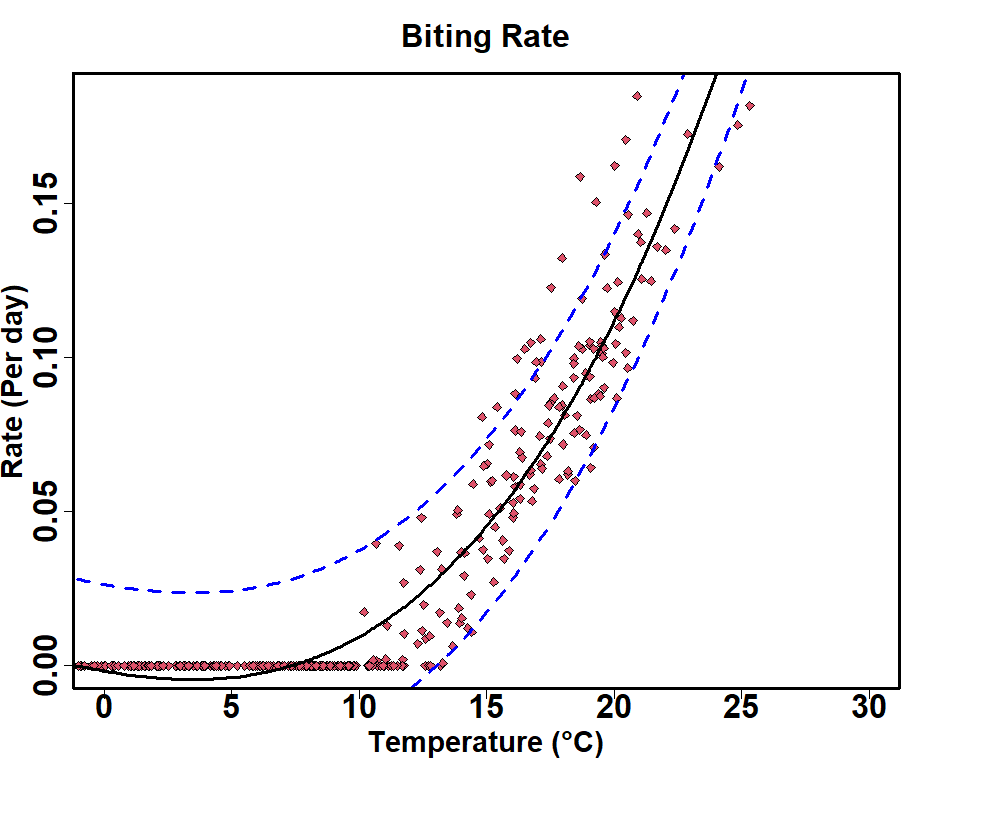 | 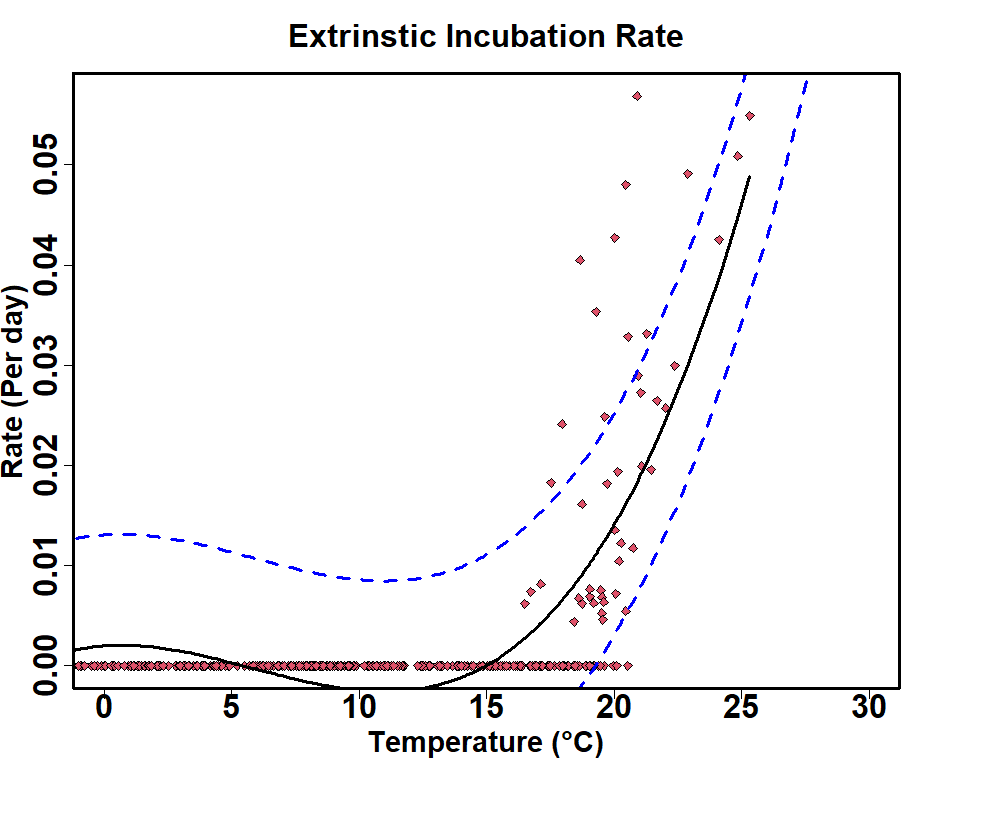 |
| **Figure S1** Thermal responses of functional traits driving mosquito population and infection rate of WNV as a function of average mean air temperature. Dashed blue lines signifies fitted distribution breath of values, solid black lines are 95% credible interval for modelled simulated response. | |

**References**

1. Ziegler, U., Lühken, R., Keller, M., Cadar, D., Van Der Grinten, E., Michel, F., Albrecht, K., Eiden, M., Rinder, M., Lachmann, L., Höper, D., Vina-Rodriguez, A., Gaede, W., Pohl, A., Schmidt-Chanasit, J., & Groschup, M. H. (2019). West Nile virus epizootic in Germany, 2018. *Antiviral Research, 162*, 39–43. https://doi.org/10.1016/j.antiviral.2018.12.005
2. Ziegler, U., Bergmann, F., Fischer, D., Müller, K., Holicki, C. M., Sadeghi, B., Sieg, M., Keller, M., Schwehn, R., Reuschel, M., Fischer, L., Krone, O., Rinder, M., Schütte, K., Schmidt, V., Eiden, M., Fast, C., Günther, A., Globig, A., … Groschup, M. H. (2022). Spread of West Nile Virus and Usutu Virus in the German bird population, 2019–2020. *Microorganisms, 10*(4), 807. https://doi.org/10.3390/microorganisms10040807

3. Ziegler, U., Santos, P. D., Groschup, M. H., Hattendorf, C., Eiden, M., Höper, D., Eisermann, P., Keller, M., Michel, F., Klopfleisch, R., Müller, K., Werner, D., Kampen, H., Beer, M., Frank, C., Lachmann, R., Tews, B. A., Wylezich, C., Rinder, M., … Lühken, R. (2020). West Nile Virus Epidemic in Germany Triggered by Epizootic Emergence, 2019. *Viruses*, *12*(4), 448. https://doi.org/10.3390/v12040448

4. Vidaña, B., Busquets, N., Napp, S., Pérez-Ramírez, E., Jiménez-Clavero, M. Á., & Johnson, N. (2020). The Role of Birds of Prey in West Nile Virus Epidemiology. *Vaccines, 8*(3), 550. <https://doi.org/10.3390/vaccines8030550>

5. Tornberg, R., & Colpaert, A. (2001). Survival, ranging, habitat choice and diet of the Northern Goshawk Accipiter gentilis during winter in Northern Finland. *Ibis, 143*(1), 41–50. <https://doi.org/10.1111/j.1474->

6. Holyoak, D. (1971). Movements and Mortality of Corvidae. *Bird Study, 18*(2), 97–106. <https://doi.org/10.1080/00063657109476300>

7. Tran, A., Fall, A. G., Biteye, B., Ciss, M., Gimonneau, G., Castets, M., Seck, M. T., & Chevalier, V. (2019). Spatial modeling of mosquito vectors for Rift Valley Fever Virus in Northern Senegal: Integrating satellite-derived meteorological estimates in population dynamics models. *Remote Sensing*, *11*(9), 1024. <https://doi.org/10.3390/rs11091024>.

8. Ezanno, P., Aubry-Kientz, M., Arnoux, S., Cailly, P., L’Ambert, G., Toty, C., Balenghien, T., & Tran, A. (2015). A generic weather-driven model to predict mosquito population dynamics applied to species of Anopheles, *Cx* and Aedes genera of southern France. *Preventive Veterinary Medicine*, *120*(1), 39–50. <https://doi.org/10.1016/j.prevetmed.2014.12.018>

9. Kerkow, A., Wieland, R., Gethmann, J. M., Hölker, F., & Lentz, H. H. K. (2022). Linking a compartment model for West Nile virus with a flight simulator for vector mosquitoes. *Ecological Modelling*, *464*, 109840. <https://doi.org/10.1016/j.ecolmodel.2021.109840>

10. Laperriere, V., Brugger, K., & Rubel, F. (2011). Simulation of the seasonal cycles of bird, equine and human West Nile virus cases. *Preventive Veterinary Medicine*, *98*(2–3), 99–110. <https://doi.org/10.1016/j.prevetmed.2010.10.013>
